# The Downward Influence of Sudden Stratospheric Warmings: Association with Tropospheric Precursors

**DOI:** 10.1175/JCLI-D-18-0053.1

**Published:** 2018-12-05

**Authors:** IAN WHITE, CHAIM I. GARFINKEL, EDWIN P. GERBER, MARTIN JUCKER, VALENTINA AQUILA, LUKE D. OMAN

**Affiliations:** The Hebrew University of Jerusalem, Institute of Earth Sciences, Edmond J. Safra Campus, Givat Ram, Jerusalem, Israel; The Hebrew University of Jerusalem, Institute of Earth Sciences, Edmond J. Safra Campus, Givat Ram, Jerusalem, Israel; Courant Institute of Mathematical Sciences, New York University, New York, New York; Climate Change zResearch Centre, University of New South Wales, Parkville, Sydney, Australia; Department of Environmental Science, American University, Washington, D.C.; NASA Goddard Space Flight Center, Greenbelt, Maryland

## Abstract

Tropospheric features preceding sudden stratospheric warming events (SSWs) are identified using a large compendium of events obtained from a chemistry–climate model. In agreement with recent observational studies, it is found that approximately one-third of SSWs are preceded by extreme episodes of wave activity in the lower troposphere. The relationship becomes stronger in the lower stratosphere, where ~60% of SSWs are preceded by extreme wave activity at 100 hPa. Additional analysis characterizes events that do or do not appear to subsequently impact the troposphere, referred to as downward and non-downward propagating SSWs, respectively. On average, tropospheric wave activity is larger preceding downward-propagating SSWs compared to non-downward propagating events, and associated in particular with a doubly strengthened Siberian high. Of the SSWs that were preceded by extreme lower-tropospheric wave activity, ~2/3 propagated down to the troposphere, and hence the presence of extreme lower-tropospheric wave activity can only be used probabilistically to predict a slight increase or decrease at the onset, of the likelihood of tropospheric impacts to follow. However, a large number of downward and non-downward propagating SSWs must be considered (>35), before the difference becomes statistically significant. The precursors are also robust upon comparison with composites consisting of randomly selected tropospheric northern annular mode (NAM) events. The downward influence and precursors to split and displacement events are also examined. It is found that anomalous upward wave-1 fluxes precede both cases. Splits exhibit a near instantaneous, barotropic response in the stratosphere and troposphere, while displacements have a stronger long-term influence.

## 1. Introduction

Approximately once every other year, the winter hemisphere westerly stratospheric polar vortex weakens, reverses in direction, and warms dramatically over the course of just a few days in a sudden stratospheric warming (SSW; see Butler et al. 2015, and references therein). Generally it is thought that such a SSW is caused by an anomalously strong upward flux of planetary waves from the troposphere (e.g., Matsuno 1971; Polvani and Waugh 2004; Sjoberg and Birner 2012). However, it is not known if the reason for this upward flux into the stratosphere is due to an anomalously large generation of wave activity in the troposphere, or due to the stratosphere being in such a state as to take advantage of the large reservoir of tropospheric wave activity and encourage anomalous wave propagation through the tropopause (Jucker 2016; Birner and Albers 2017; de la Cámara et al. 2017). Because of the hemispherical differences in topography, all but one of the observed SSWs have occurred in the Northern Hemisphere (NH) (e.g., Charlton and Polvani 2007).

It is acknowledged that SSWs can have an appreciable influence on the tropospheric circulation below for up to 2 months following the onset of the event (e.g., Baldwin and Dunkerton 2001; Nakagawa and Yamazaki 2006; Mitchell et al. 2013; Hitchcock and Simpson 2014; Kidston et al. 2015). In particular, SSWs on average precede a persistent equatorward shift of the North Atlantic eddy-driven jet [i.e., a negative phase of the North Atlantic Oscillation (NAO)]. The eddy-driven jet is collocated with the extratropical storm tracks, and hence plays a crucial role in determining the weather over North America and Europe (e.g., Kidston et al. 2015). Additionally, it has been shown that SSWs result in an increase in cold-air outbreaks in the midlatitude NH (Thompson et al. 2002; Tomassini et al. 2012) as well as high-latitude blocking events (Martius et al. 2009). Thus, it has been suggested that the skill of tropospheric seasonal forecasts can be improved by enhancing our understanding of SSWs and their downward influence on the tropospheric circulation (Marshall and Scaife 2010; Scaife et al. 2012; Smith et al. 2012; Sigmond et al. 2013; Tripathi et al. 2015).

While there is a clear aggregate impact of SSWs on the troposphere, there is considerable variation between individual events (Baldwin and Dunkerton 2001; Sigmond et al. 2013). Indeed, some events exhibit no visible impact and hence this has led to studies defining SSWs as either “downward” (DW) or “non-downward” (NDW) propagating (Jucker 2016; Kodera et al. 2016; Runde et al. 2016; Karpechko et al. 2017). However, there is debate about whether there is an actual DW communication of information from the stratosphere, or whether the observed influence is related to variability inherent to the troposphere (Kidston et al. 2015).

Previous studies have highlighted the role of the stratosphere in determining the extent of the DW influence. It has been suggested that the type and magnitude of the wave forcing (be it wave 1 or wave 2) entering the stratosphere (e.g., Nakagawa and Yamazaki 2006), the type of SSW (split or displacement) that occurs (e.g., Mitchell et al. 2013; S13; O’Callaghan et al. 2014; Seviour et al. 2016), the depth to which the initial warming descends in the stratosphere (Gerber et al. 2009; Hitchcock et al. 2013), and the persistence of the SSW in the lower stratosphere (Hitchcock and Simpson 2014; Maycock and Hitchcock 2015) can all play a role, either individually or collectively, in determining the tropospheric response. For instance, Nakagawa and Yamazaki (2006) found that observed SSW events that were followed by a significant long-lasting tropospheric anomaly were associated with an enhanced upward flux of wave 2. Mitchell et al. (2013) and Seviour et al. (2013) found that the observed tropospheric response was dependent on the SSW type; split SSWs were associated with such a response, whereas displacement SSWs were not. Recently, using a large compendium of modeled SSWs, Maycock and Hitchcock (2015) found only small differences between both types, but also found that the surface responses were not robust to the algorithm used to classify the events. They also suggested that the tropospheric impact was dependent on whether the lower-stratospheric circulation anomalies persisted, a point that was also proposed by Hitchcock and Simpson (2014) and Karpechko et al. (2017) using reanalysis data and a full chemistry–climate model, as well as by Jucker (2016) using idealized GCM experiments. Lehtonen and Karpechko (2016) and Karpechko et al. (2017) both indicated the role of enhanced upward-propagating planetary waves prior to the onset of the SSW as well as its continuation for a up to a week after the onset.

On the other hand, both observational and modeling studies have suggested that the troposphere may play a role in the initial forcing of some SSW events (e.g., Martius et al. 2009; Garfinkel et al. 2010; Cohen and Jones 2011; Dai and Tan 2016; Hitchcock and Haynes 2016; Bao et al. 2017) as well as the ensuing tropospheric response, be it due to the state of the troposphere prior to the onset (Black and McDaniel 2004) or to the presence of synoptic-scale eddy feedbacks (Limpasuvan et al. 2004; Song and Robinson 2004; Domeisen et al. 2013; Hitchcock and Simpson 2014). However, while precursors such as blocking events have been found to occur before 25 of the 27 SSWs observed in ERA-40 (Martius et al. 2009), only 6% of blocking events during 1957–2001 were actually followed by an SSW. These results indicate that tropospheric precursors are perhaps not a useful predictor, despite them occurring prior to many SSWs. Garfinkel et al. (2010) found that surface variability over the North Pacific and eastern Europe could either deepen or flatten the troughs and/or ridges associated with tropospheric stationary planetary waves. Such precursors over these two regions then lead to changes in the upward wave flux and possibly the onset of a weaker polar vortex, followed by its DW propagation. Depending on the magnitude and spatial location of this anomalous forcing, either a split or displacement SSW may occur (e.g., Cohen and Jones 2011). Further, Black and McDaniel (2004) observed that the determination of the DW propagation of a SSW depended on the pre-existing tropospheric state; in the case of NDW-propagating events, the troposphere was already in a positive northern annular mode (NAM)-like state that acted to mask the DW stratospheric influence. In the case of DW-propagating events, the troposphere was already in a negative NAM-like state, although slightly out of phase, latitudinally, with the canonical NAM.

In contrast, modeling studies by Gerber et al. (2009) and Hitchcock and Simpson (2014) suggest that differences between DW and NDW events are associated primarily with differences in tropospheric variability. That is to say, they hypothesize that there is a deterministic influence of SSWs on the troposphere (a forced response), which is combined with an essentially stochastic component associated with internal tropospheric variability. The latter can mask or enhance the DW forced signal, and thus predicting the response to a SSW will likely be limited by our ability to forecast tropospheric weather. This also speaks to the difficulty in being able to understand the mechanisms behind the DW propagation of a SSW.

One of the key aims of this paper is to identify and determine the robustness of tropospheric precursory features to SSWs as well as to assess whether these tropospheric precursors may be important for discriminating between DW and NDW SSWs, using a large compendium of SSWs obtained from the Goddard Earth Observing System Community Climate Model (GEOSCCM). The paper then has the following structure: in section 2 we present a description of the GEOSCCM model integrations used in this study, and of the methods used to identify SSWs (Charlton and Polvani 2007, hereafter CP07) and split and displacement vortex events (Seviour et al. 2013, hereafter S13), and also determine whether these events are DW or NDW propagating (Jucker 2016; Runde et al. 2016; Karpechko et al. 2017); in section 3 we present the results; and finally in section 4 we present a summary and discussion.

## 2. Methodology

### a. Model output

We utilize a series of model integrations that were performed using the Goddard Earth Observing System Chemistry–Climate Model, version 2 (see Rienecker et al. 2008). The GEOSCCM couples the GEOS-5 (Molod et al. 2012) atmospheric general circulation model (GCM) with StratChem, a comprehensive stratospheric chemistry module (Pawson et al. 2008). In total, 40 historical-run integrations are here analyzed, 25 of which are 30 years in length (January 1980 to December 2009) and 15 are 55 years in length (January 1960 to December 2014), which yields a total of 1575 years of data to analyze. These are described in more detail in Garfinkel et al. (2015), Aquila et al. (2016), and Garfinkel et al. (2018). The integrations were performed for different purposes and therefore this “super ensemble” encompasses a range of forcings and physical parameterizations. These include changing sea surface temperatures, sea ice, and greenhouse gas concentrations, as well as ozone-depleting substances, solar variability, and volcanic eruptions. We note that there is a slight influence of SSTs on the DW and NDW propagation of SSWs with there being slightly more DW SSWs than NDW SSWs during El Niño years, but it is comparatively weak and this will be discussed in a future publication. We also note that the two different time periods (i.e., pre- and post-satellite era) over which the integrations are run do not have an influence on the results. The model was run using 72 vertical layers with a lid at 0.01 hPa, although we base our analysis on 14 levels ranging from 700 hPa up to 1 hPa. We note that at 700 hPa there were small areas over mountain regions for which no value was outputted from the model; these were filled in using an interpolation scheme in this study so that we could decompose the heat flux into different zonal wavenumbers. The horizontal resolution is 2° latitude by 2.5° longitude.

### b. SSW definitions

To define SSW events in the GEOSCCM model integrations described above, we first utilize a simplified version of the World Meteorological Organization (WMO) criteria proposed by CP07 where SSWs are defined by a reversal of the zonal-mean zonal wind *ū* at 60°N and 10 hPa to easterly winds from 1 November to 31 March. This criterion is supplemented by the requirement that winds return to a westerly state for a period of 10 consecutive days prior to 30 April, which helps avoid counting any final warmings, and a separation of at least 20 days between two consecutive events, to avoid counting the same SSW event twice (see also the corrigendum of CP07). Using the SSW definition above, a total of 962 SSWs (see Table 1) are found giving a ratio of 0.61 per year, a ratio a little smaller than that found in observations [also see Table 1 in Butler et al. (2015)]. We note that this slight decrease in the SSW frequency relative to that observed may be due to the fact that the climatological planetary-wave flux entering the stratosphere near 100 hPa in our 40 runs is smaller than in ERA-Interim.

We also identify the two characteristic types of extreme vortex variability—split and displacement SSWs—using the 2D moment analysis method described by S13. In particular, the geopotential height *Z* at 10 hPa, rather than the potential vorticity as in Mitchell et al. (2013), is used in this method. S13 detail this method, but there are three parameters that are modified for this study. The first is the edge of the polar vortex, which we here define as the December–March (DJFM) climatological mean *Z* at 60°N and 10 hPa [as in Maycock and Hitchcock (2015)], where the climatology is defined as the average during DJFM in all 40 ensemble members. The second and third are the thresholds for the split and displacement SSWs, which depend on the values of the centroid latitude and aspect ratio. We here choose the thresholds as the most equatorward 5% of centroid latitudes and largest 5% of aspect ratios in all ensemble members, yielding thresholds of 64.38°N and 2.074 respectively (compare these values to the respective 5.7%/66°N and 5.2%/2.4 used in S13). We note that the results are not sensitive to slight changes in the thresholds used here. We also note that a handful of events satisfy both criteria, in which case they are marked as unclassifiable, to try and best ensure independent events. Using this method, we find a total of 903 events with 400 splits, 500 displacements, and 3 unclassified (see Table 1). Note that these events are not the same as the 962 SSW events identified using the CP07 method, as we do not here classify the CP07-identified SSWs as splits or displacements. Nevertheless, 545 of the CP07-identified SSWs overlap within ±10 days of an identified displacement or split SSW.

### c. DW- and NDW-propagating event definitions

To define whether a given event is DW or NDW propagating we utilize the NAM index. In this study we compute a simplified NAM index based on the polar-cap average geopotential height *Z* (Baldwin and Thompson 2009). Standardized *Z* anomalies are calculated at each level as the deviation from the 60-day low-pass filtered daily climatology, which are subsequently smoothed using a 3-day running mean, following Martineau and Son (2015), although we note that quantitatively similar results can be found using different filtering windows. The anomalies are then area-averaged (i.e., multiplied by cos*φ* where *φ* is latitude) over 60°–87°N, divided by the standard deviation at each level, and multiplied by −1 so that, as is conventional, a negative NAM index identifies with a positive *Z* anomaly and vice versa.

Four definitions have been proposed recently to characterize the DW propagation of SSWs using the NAM index: one by Runde et al. (2016), two by Jucker (2016), and one by Karpechko et al. (2017). In this manuscript we mostly present results using that by Karpechko et al. (2017) and hence this is the one we briefly summarize here. The descriptions of the other three are included in the online supplemental material. Karpechko et al. (2017) introduced three criteria that must be satisfied, these being that 1) the averaged NAM index at 1000 hPa over the period ranging from 8 days until 52 days after the onset date must be negative, 2) the fraction of days in this 45-day period on which the NAM index at 1000 hPa is negative must be greater than 0.5, and 3) the fraction of days in this 45-day period on which the NAM index at 150 hPa is negative must be greater than 0.7. Note that for the first two criteria we use the NAM at 850 hPa to reduce complications with topography and for the third we use 100 hPa to ensure that the anomalies persist in the lower stratosphere, although we note that the results are not sensitive to the choice of level. These criteria are chosen to ensure that there is a long-lasting tropospheric signal of the negative NAM anomalies associated with the upper-tropospheric/lower-stratospheric negative anomalies. See Table 1 for the numbers of DW and NDW SSWs resulting from all four DW definitions.

## 3. Results

We start by identifying apparent precursory features to SSWs (both DW- and NDW-propagating) using composites over all of the modeled SSW events. We then test the robustness of these precursors using different DW definitions as introduced in section 2 and random composites of tropospheric events, before examining the number of SSWs that are actually preceded by these precursors. Finally, we briefly examine the precursory features to splits and displacements along with their division into DW and NDW events. Note that herein we define a precursor to be an anomalous feature that is found to occur prior to a SSW event, but do not claim there to be any deterministic aspect, as there is no one-to-one relationship between any of the precursors we identify and the subsequent stratospheric state due to the large internal variability of the stratosphere.

### a. Composite analyses of DW and NDW events

**FIG. 1.**
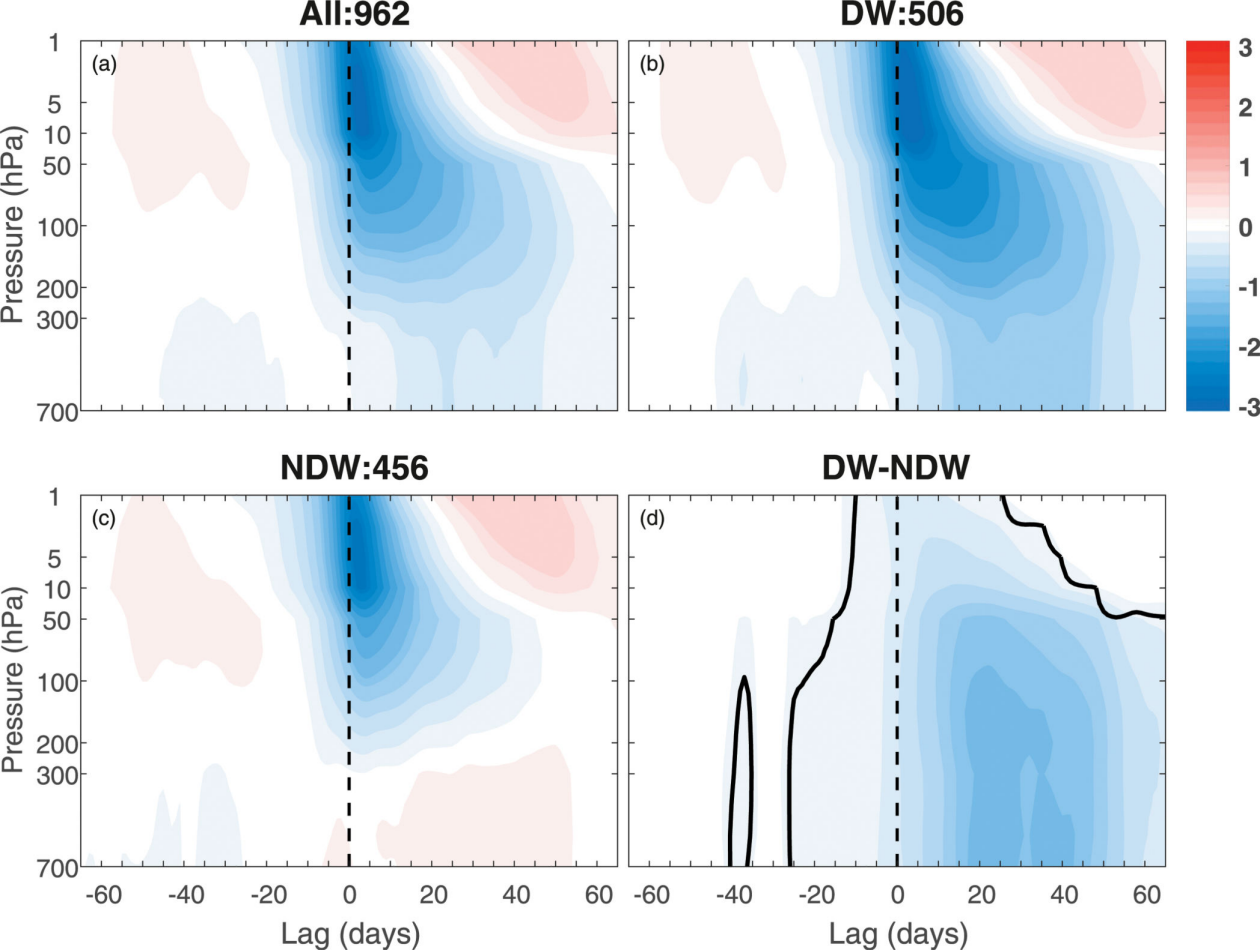
The composite evolution of the NAM index for (a) all SSWs calculated in the entire ensemble of integrations and (b) DW-propagating SSWs calculated using the Karpechko et al. (2017) criteria (see our section 2c); (c) as in (b), but for NDW-propagating SSW events; and (d) the composite difference between the DW- and NDW-propagating events (DW–NDW). The units are in standard deviations. The thick black line in (d) represents statistical significance at the 95% level.

As a starting point, we examine the evolution of the NAM index, which has been traditionally used as a measure of stratosphere–troposphere coupling. The NAM for all SSWs is composited at lag zero according to the onset date of the SSW (see section 2). We only show results using the DW definition of Karpechko et al. (2017) but note that the robustness of these results to DW definition is discussed in section 3b. Figure 1 shows the NAM index composited over all SSW events in all of the ensemble members (Fig. 1a) (a total of 962; see Table 1), all DW-propagating SSW events (Fig. 1b) (506; as determined by the criteria in section 2), and all NDW-propagating SSW events (Fig. 1c) (456), as well as the composite difference between the DW- and NDW-propagating events (Fig. 1d) (hereafter DW – NDW). In the all event composite (Fig. 1a), the NAM index is similar to the canonical “dripping-paint” pattern first highlighted by Baldwin and Dunkerton (2001). The negative anomalies initialize around lags −15 to −10 above ~250 hPa, and at lag zero maximize in the upper stratosphere. The negative anomalies propagate DW to the lower stratosphere over the next few weeks and start to recover in the upper stratosphere after lag +20, although those in the lower stratosphere persist until lag +60. Negative anomalies are visible in the troposphere for all positive lags, but with much smaller amplitude than those in the stratosphere.

Upon subdividing the total into DW- and NDW-propagating events (Figs. 1b,c), it can be seen that the DW events have a much stronger influence on the troposphere after lag 0, by construction, with negative NAM anomalies reaching down to near the surface and persisting for over 60 days. At positive lags, the DW composite (Fig. 1b) has magnitudes of around twice that of the total (Fig. 1a) in the troposphere, which is due to the cancellation between the negative DW anomalies and the weakly positive NDW anomalies in Fig. 1c. Further, the magnitude of the negative anomalies in the upper stratosphere is larger for the DW events, and those in the lower stratosphere persist for considerably longer during DW events. Finally, there are larger negative tropospheric anomalies in the DW composite compared to the NDW composite prior to lag zero. Zonal-mean anomalies prior to lag zero have been found with both the same sign (Jucker 2016; Karpechko et al. 2017) and also with opposite sign (Hitchcock and Haynes 2016) using a large compendium of modeled SSWs. To this point, Gerber et al. (2010) showed such precursor anomalies to be model-dependent as well as configuration-dependent. For instance, Gerber et al. (2010), using the Canadian Middle Atmosphere Model (CMAM) found such precursors, but using a slightly different model configuration, Hitchcock and Simpson (2014) did not. It appears that DW SSW events appear to be stronger in overall magnitude in both the troposphere and stratosphere, persist for longer in the lower stratosphere, and have evidence of tropospheric preconditioning, in comparison to those that are NDW propagating.

**FIG. 2.**
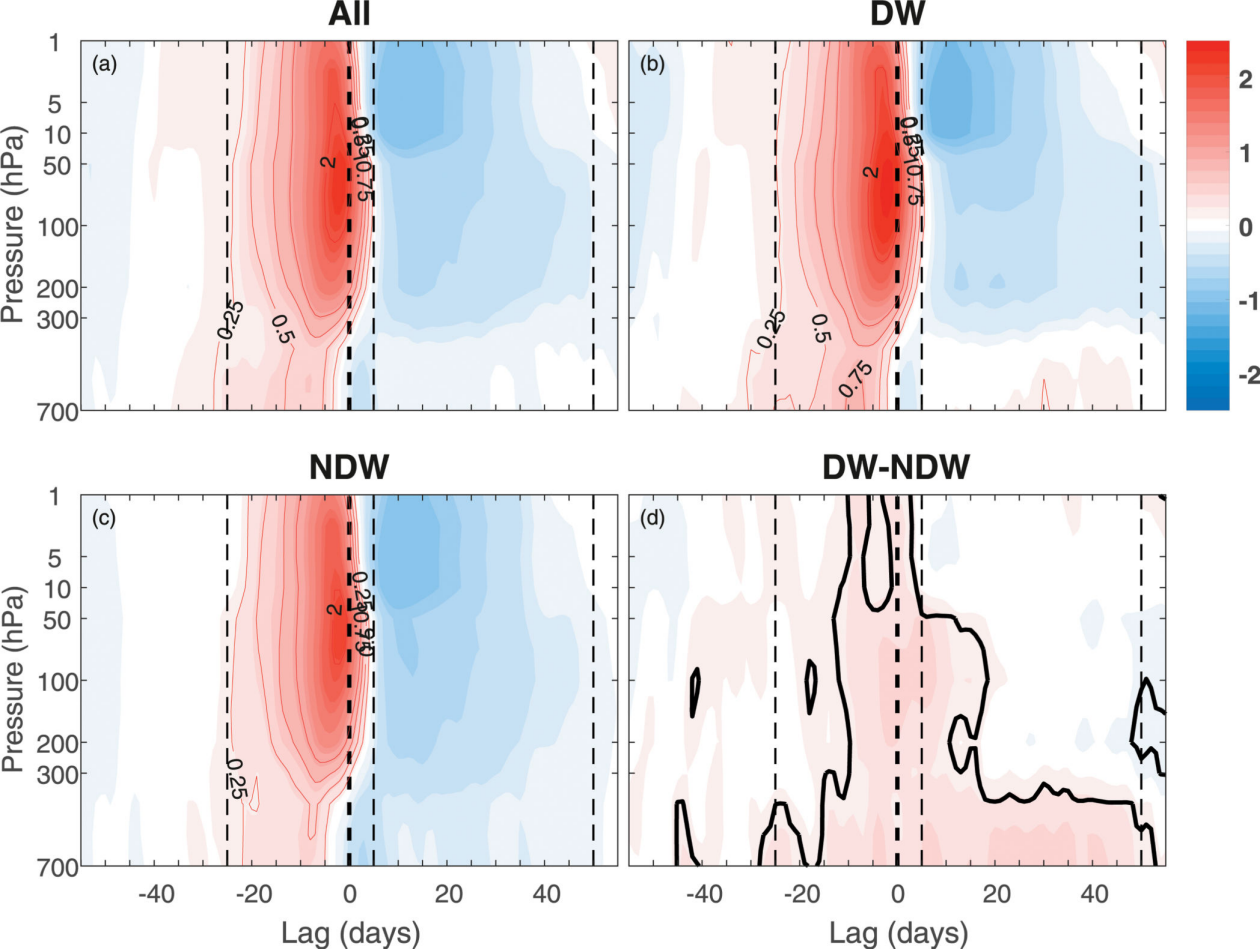
As in Fig. 1, but for the anomalous vertical component of the Eliassen–Palm flux, *F*^(*z*)^ (see text), averaged over the latitude band of 45°–75°N and filtered for planetary waves 1 and 2. Note that *F*^(*z*)^ has been scaled by the climatological standard deviation at each level so that the contours have units of standard deviation. Certain positive-valued contours have been added to aid in the discussion. The dashed vertical lines represent the start and end of the different lag stages used throughout the remainder of the manuscript (see text). The dashed line corresponding to zero lag has a double thickness for clarity. The thick black line in (d) represents statistical significance at the 95% level.

To examine the differences in upward wave activity between DW and NDW events, in Fig. 2 we show the height–time evolution of the vertical component of the Eliassen–Palm (EP) flux

(1) $$F^{\left(z\right)}=\rho_{0}a\cos\varphi \left(\left[f-\frac{1}{a\cos\varphi }{\left(\bar{u}\cos\varphi \right)}_{\varphi }\right]\bar{v^{\prime }\theta^{\prime }}/{\bar{\theta }}_{z}-\bar{w^{\prime }u^{\prime }}\right)$$

(Andrews and McIntyre 1978; Andrews et al. 1987), where *φ* and *z* are the latitude and log-pressure height coordinates; *u*, *v*, and *w* are the zonal, meridional, and vertical components of the wind; *θ* is the potential temperature; *f*, *a*, and *ρ*_0_ are respectively the Coriolis parameter, Earth’s radius, and basic-state density; and overbars and primes represent the zonal mean and deviations from the zonal mean, respectively. The term *F*^(*z*)^ is averaged over the latitude band of 45°–75°N and filtered for planetary waves 1 and 2, and as in Fig. 1, presented as composites over all SSWs (Fig. 2a), DW SSWs (Fig. 2b), NDW SSWs (Fig. 2c), and the DW – NDW difference (Fig. 2d). As advocated by Jucker (2016) and Birner and Albers (2017), the anomalies are standardized by dividing each level by the climatological standard deviation so that, for example, a value of 2 represents two standard deviations from the mean. This allows one to determine how strong the wave bursts at a given level are, compared to general variability at that level (Jucker 2016; Birner and Albers 2017). Prior to the onset date, it is clear that in the total, DW, and NDW composites, the anomalous wave flux at stratospheric levels is, in a relative sense, larger than at tropospheric levels. In particular, in the DW composite, the anomalies have a magnitude of nearly 2.5 standard deviations in the stratosphere and of 0.75 standard deviation in the troposphere, whereas in the NDW composite, the values are comparatively small with values of 2 and 0.25 standard deviations in the stratosphere and troposphere. The gradual upward propagation at negative (−30 to −15) lags also hints that for some events, there is a tropospheric source of wave activity that may well be amplified in the stratosphere closer to the onset date. The DW–NDW composite makes clearer the significant differences with values of around 0.25–0.5 standard deviations, becoming largest in the stratosphere closer to the onset date.

At positive lags, the anomalies in both the DW and NDW composites are negative in the stratosphere, indicating reduced upward wave propagation after the onset date. However, we note that the positive anomalies around the onset date do persist in the stratosphere for up to a week. In the troposphere, the anomalies are of opposite sign between DW and NDW events; for the DW events, there are weakly positive anomalies (in this standardized sense; if using the full field then they become larger), which we note are dominated by wave 2, whereas for NDW events there are negative anomalies. The weakly positive anomalies for DW events are seemingly in disagreement with Hitchcock and Simpson (2014) and Hitchcock and Haynes (2016), who found reduced vertical wave flux during the recovery phase, but since they are of very small magnitude compared to tropospheric variability, we do not expect the difference between this feature and the afore-mentioned studies to be significant. We also note that synoptic waves contribute in the troposphere at positive lags (not shown).

These *F*^(*z*)^ anomalies allow us to define certain lag stages in the evolution of the DW and NDW SSWs (see dashed vertical lines). The first is the preconditioning stage (PC) from lags −25 to −1, and these lags are chosen as they represent the approximate duration of the significant tropospheric precursor DW–NDW differences, although we note that the tropospheric and stratospheric anomalies intensify at around lag −15. The second is the onset stage (ONS) from lags 0 to +5, which is associated with continued (reduced) anomalous upward wave propagation in the stratosphere (troposphere). Finally, we classify the recovery stage (REC) over lags +6 to +50, which represents the approximate time scale over which the tropospheric DW–NDW differences disappear. Note that results in this paper are not sensitive to slight changes in the definition of these lags.

**FIG. 3.**
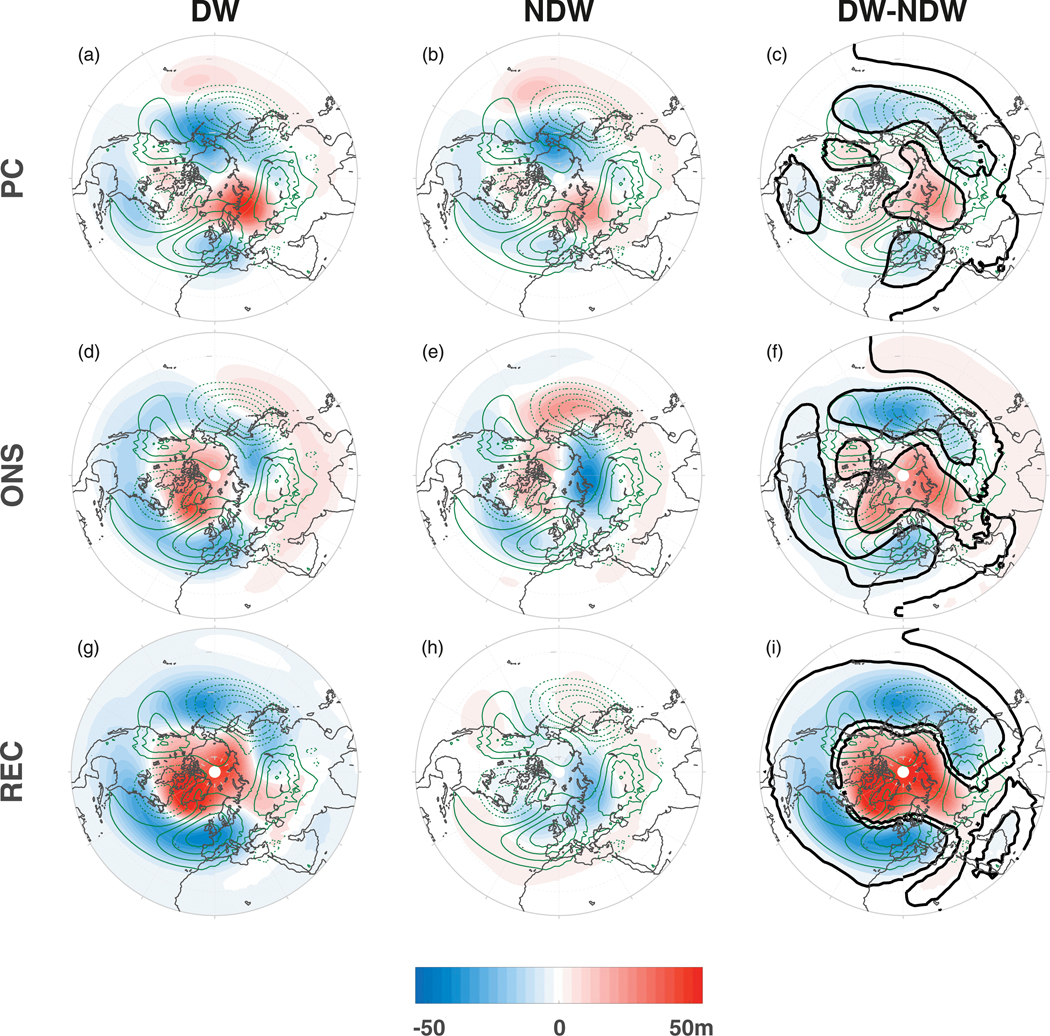
Geopotential height *Z* anomalies (shading; units m) at 700 hPa, averaged over the (a)–(c) PC stage, (d)–(f) ONS stage, and (g)–(i) REC stage, and composited over (left) DW events and (middle) NDW events, and (right) DW – NDW differences. Green contours show the November–February climatology calculated as the average over all of the 40 experiments with a contour interval of 25 m starting at 15 m. The thick black line is as in Fig. 1.

With the zonal-mean NAM precursors in mind (Fig. 1), we now determine if there are any such precursors in a latitude–longitude sense. In Fig. 3 we show *Z* anomalies at 700 hPa averaged over the PC stage (top row), ONS stage (middle row), and REC stage (bottom row). The November–February climatology for each variable is superimposed as green contours and we note that the climatologies in these GEOSCCM integrations agree well with observations (e.g., Garfinkel et al. 2010).

In the PC stage, the *Z* anomalies for the DW (Fig. 3a) and NDW (Fig. 3b) composites show similar spatial patterns, with a clear wave-1-like structure consisting of negative anomalies northward of 60°N over the North Pacific and positive anomalies over Scandinavia and Europe. These negative (positive) anomalies project onto the climatological stationary planetary wave-1 centers of action, albeit slightly offset to the northeast (northwest), respectively. In the DW composite, the magnitudes of the anomalies are noticeably larger than in the NDW composite; in particular, the positive anomalies over northern Europe are doubled in the DW composite. This difference in magnitudes is highlighted in the DW–NDW composite (top right) with negative and positive differences over the Aleutian low sector and the Siberian high sector respectively. We also note the regions of positive and negative anomalies farther equatorward over the North Pacific and North Atlantic respectively. Over the North Atlantic, the anomalies are significantly more negative for the DW events.

During the ONS stage (middle row), positive anomalies appear over the polar cap with an annulus of negative anomalies starting to develop at midlatitudes for the DW events. For the NDW events however, positive and negative anomalies develop over the Aleutian low and Siberian high regions, respectively, projecting negatively onto the climatological centers and suggesting a reduced upward wave-1 flux. This yields differences that still show a wave-1 pattern over the North Pacific and Siberia, along with more widespread negative differences over the North Atlantic (compared to during the PC stage). The latter highlights the canonical DW influence of SSWs. The NAM at lags 0 to +5 is not utilized in the Karpechko et al. (2017) DW definition and hence these anomalies are not forced by the averaging associated with the definition. During the REC stage (bottom row), the strongest anomalies are associated with the DW events (indeed, with much smaller anomalies in the NDW composite), which exhibit a highly zonal pattern, with positive anomalies at high latitudes surrounded by an annulus of negative anomalies at midlatitudes, projecting onto the negative phase of the NAO. While the annulus pattern during REC is present by construction, the DW–NDW difference during the PC and ONS stages is not.

**FIG. 4.**
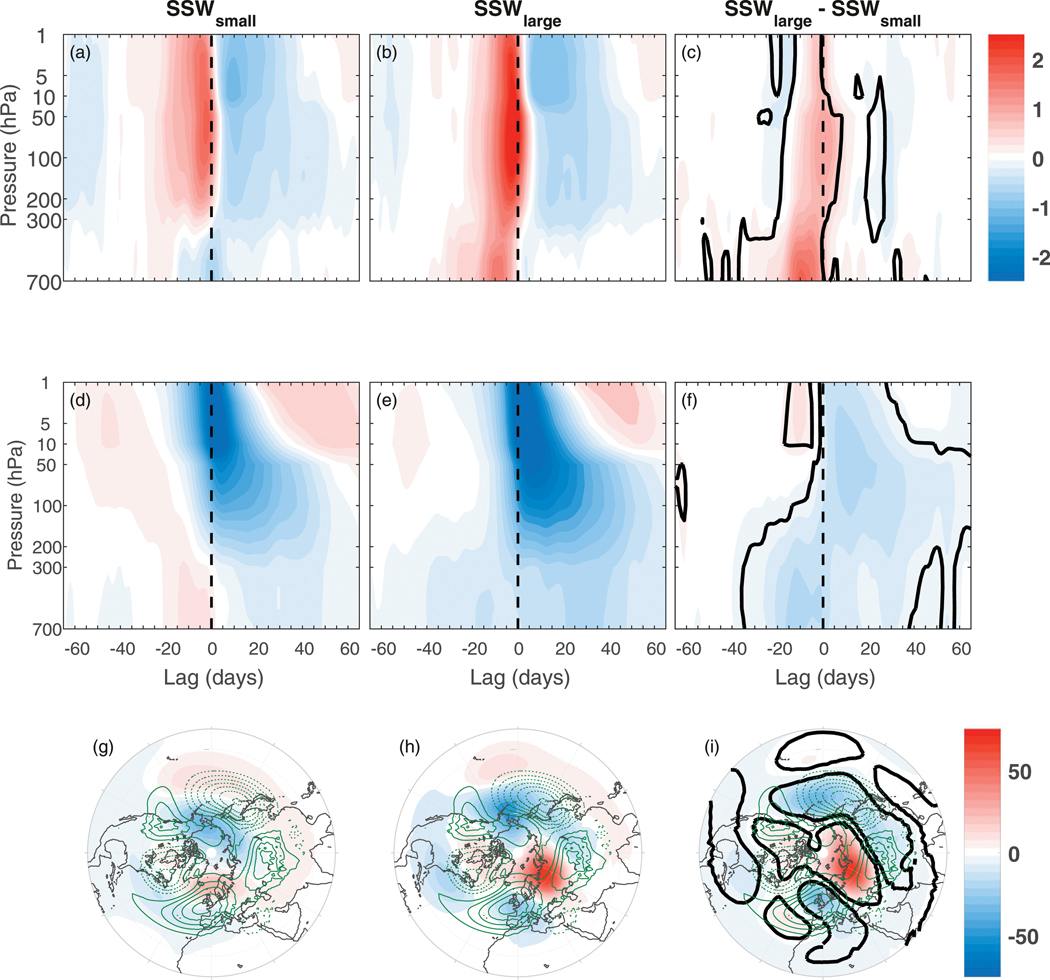
Composites of (a)–(c) *F*^(*z*)^ (filtered for waves 1–2 and standardized as in Fig. 2), (d)–(f) NAM, and (g)–(i) *Z* stratified according to the strength of *F*^(*z*)^ at lags −15 to −1 at 500 hPa. Shown are (left) the *F*^(*z*)^, NAM, and *Z* for the half of SSWs with the smallest *F*^(*z*)^ anomalies (SSW_small_), (middle) the half of SSWs with the largest *F*^(*z*)^ anomalies (SSW_large_), and (right) show the corresponding SSW_large_ – SSW_small_ differences. Thick black lines in the right column are as in Fig. 1. Green contours in the bottom row are as in Fig. 3.

In the previous three figures, there is clearly on average, enhanced upward wave activity in the troposphere, a more negative tropospheric NAM and an enhanced Siberian high for DW events prior to the SSW onset. We now further examine the connection between these three features in Fig. 4, but instead of splitting the SSWs according to the sign and magnitude of the NAM after the onset (as in Figs. 1–3), we split them according to the strength of *F*^(*z*)^ (filtered for waves 1–2) in the lower troposphere, before the onset date. In particular, we composite the SSWs into the half of SSWs with the smallest *F*^(*z*)^ at 500 hPa, averaged over lags −15 to −1 (SSW_small_) (shown in the left column) and the half of SSWs with the largest such *F*^(*z*)^ (SSW_large_) (shown in the middle column). In the right column, the SSW_large_ – SSW_small_ differences are then shown. In the top row, the clear feature is the larger *F*^(*z*)^ anomalies throughout the troposphere and stratosphere at negative lags in SSW_large_ events, although note that the lower-tropospheric anomalies at negative lags are by construction.

In the middle row (the NAM index), it is clear that the tropospheric NAM is more negative for SSW_large_ events at both negative and positive lags as well as being more negative in the stratosphere after the onset. Finally, in the bottom row (*Z*), the clearest differences between the SSW_large_ and SSW_small_ events are the negative and positive anomalies over the North Pacific and Siberian high regions, respectively, which are much enhanced for the SSW_large_ events. These project positively onto the climatological centers of action, and thus are likely linked with the enhanced *F*^(*z*)^ seen in the top row. Together with the *F*^(*z*)^ panels, the NAM and *Z* anomalies suggest that enhanced upward lower-tropospheric wave activity prior to the SSW onset date may lead to a weaker polar vortex and subsequently be associated with a more negative tropospheric NAM after the onset.

**FIG. 5.**
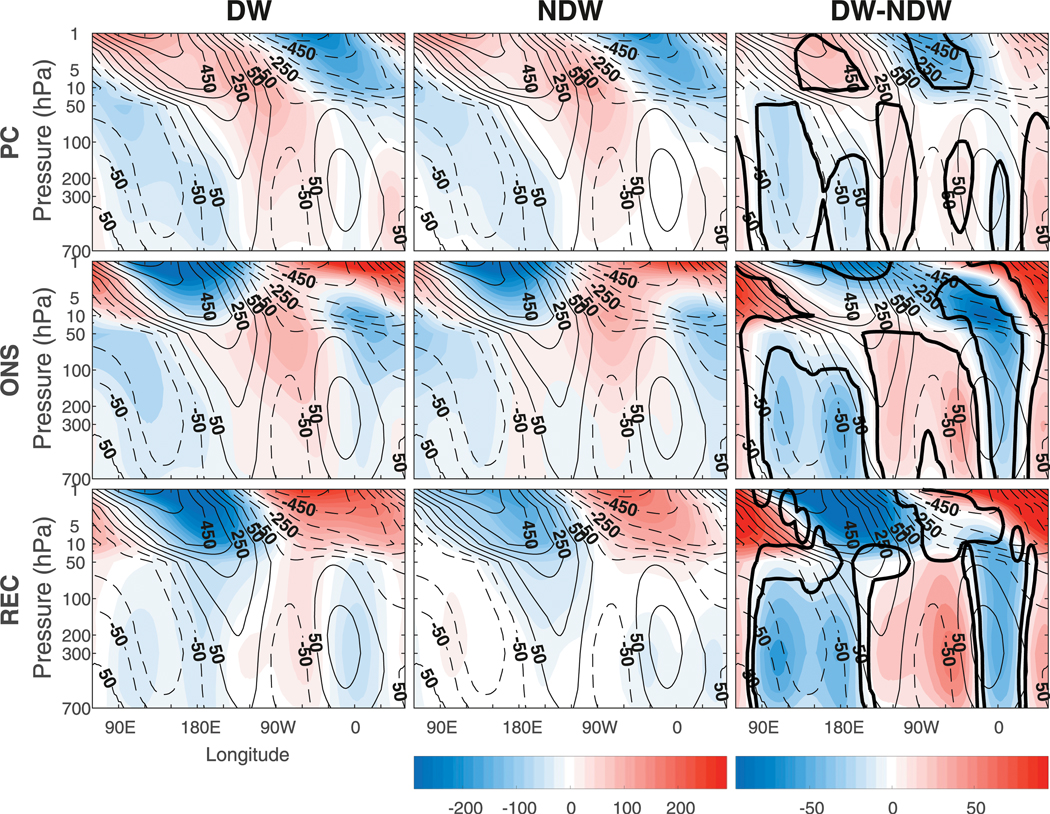
As in Fig. 3, but for the longitude–height cross sections of *Z*ʹ (i.e., deviation from the zonal mean) averaged over the latitude band 50°–60°N. The units are in m. Thin black contours show the November–February climatology calculated as the average over all of the 40 experiments with contours at −650, −550, … , 550, 650 m.

To determine the vertical extent of the *Z* anomalies, we show longitude–height cross sections of *Z*ʹ (i.e., the deviation from the zonal mean) in Fig. 5, averaged over the same lag stages as in Fig. 3 and over the latitude band of 50°–60°N. This latitude band is chosen as it best captures the negative and positive anomalies over the Aleutian low and Siberian high regions shown in Fig. 3. In the climatology (thin black contours), there is a clear westward tilt with height of *Z*ʹ agreeing with the well-known westward tilt of upward-propagating planetary waves (e.g., Andrews et al. 1987). Note that *Z*ʹ has a wave-1 structure in the stratosphere with one ridge and one trough, but is associated with higher wavenumbers in the troposphere (multiple ridges and troughs). This agrees with the Charney–Drazin criterion (Charney and Drazin 1961), which states that only planetary waves can propagate into the stratosphere and smaller-scale waves are limited to propagation in the troposphere.

During the PC stage (Fig. 5, top row), the anomalies for both DW and NDW events project positively onto the climatological *Z*ʹ anomalies and exhibit the canonical westward tilt with height, indicating anomalous upward wave propagation from the troposphere to the lower-to-middle stratosphere. In particular, in the troposphere, there are negative anomalies spanning from 70°E eastward to ~150°W, and positive anomalies from 150°W eastward to ~60°E. These agree with the *Z*ʹ anomalies at 700 hPa shown in Fig. 3. In the difference plot, it is clear that the anomalies associated with DW events are generally larger in magnitude, indicating enhanced upward wave propagation.

After the onset date (Fig. 5, middle row), the anomalies above 10 hPa change sign, thus projecting negatively onto the climatological centers. This is likely associated with reduced upward wave propagation deep into the stratosphere after a SSW event, in agreement with the Charney–Drazin criterion. Below 50 hPa, the anomalies and differences look generally similar to during the PC stage although they are slightly more connected, suggesting continued upward wave propagation into the lower stratosphere. During the REC stage (Fig. 5, bottom row), the upper-to-middle stratospheric anomalies extend deeper into the lower stratosphere compared to during the ONS stage and are still of opposite sign to the climatology. The latter point indicates that waves are absent above 50 hPa under DW events, and much reduced under NDW events. This is in agreement with a SSW event that has a more negative NAM (Fig. 1). Below 50 hPa, the GPH anomalies lose their westward tilt with height, instead either exhibiting more of an eastward tilt, particularly over the North Pacific (Fig. 5g), or vanishing almost entirely (Fig. 5h).

**FIG. 6.**
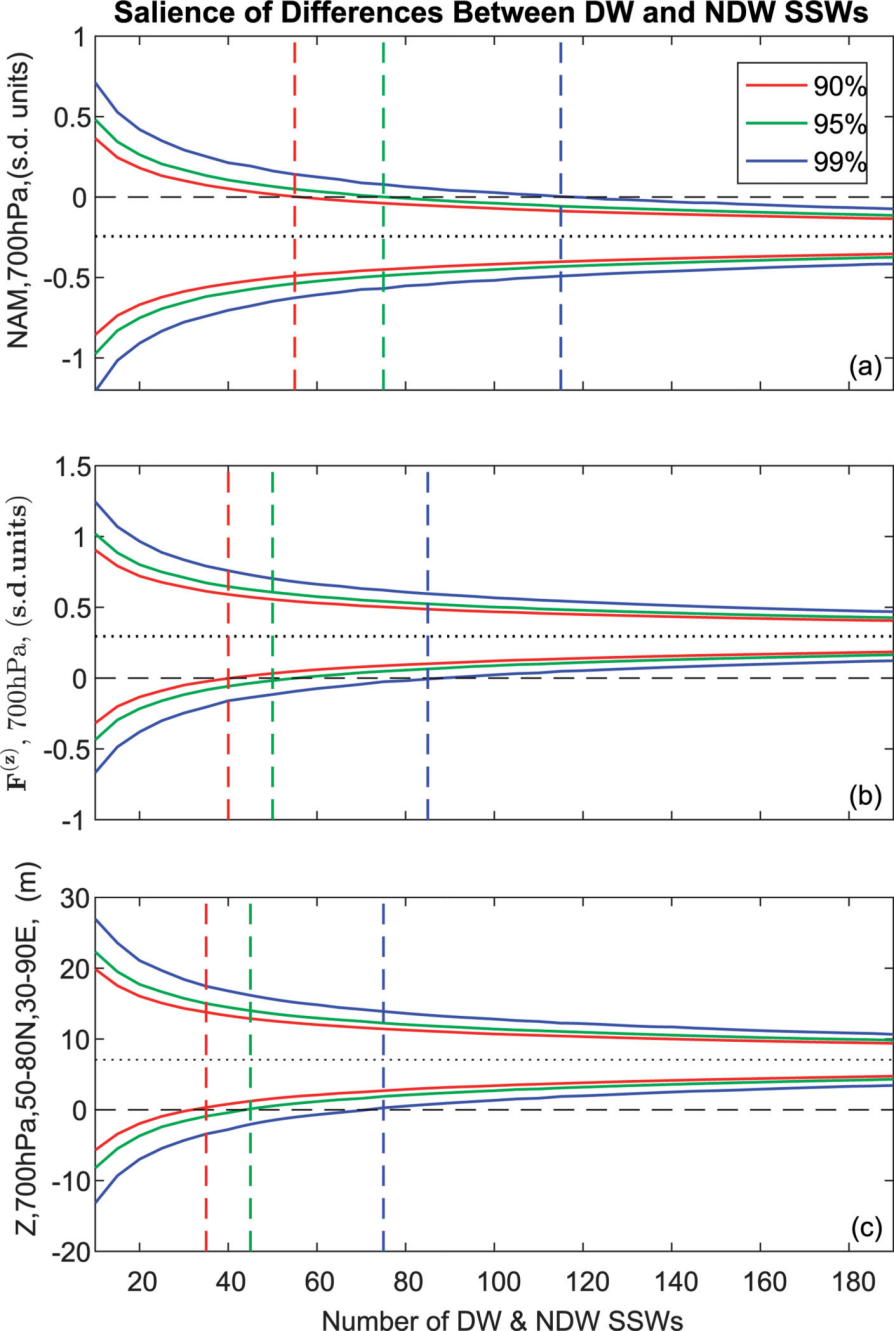
Confidence intervals for the difference (DW – NDW) of (a) the NAM index averaged over lags −25 to −1 and at 700 hPa, (b) *F*^(*z*)^ anomalies at 700 hPa filtered for waves 1–2 and area averaged over 45°–75°N, and (c) *Z* anomalies at 700 hPa averaged over 50°–80°N, 30°–90°E and over lags −25 to −1. The confidence intervals are estimated using a Monte Carlo simulation of 100 000 repetitions for different sample sizes ranging from 10 to 455. The red, green, and blue curves represent the 90%, 95%, and 99% confidence intervals, and the respective colored vertical dotted lines represent the sample size for which the upper bound crosses zero (indicated by the dashed black line). The dotted black line represents the overall DW–NDW composite over all DW and NDW events, as shown in Figs. 1–3, respectively.

It is worthwhile to examine how many SSWs are required to find precursory features such as those found in Figs. 1–5. For instance, these precursor features to DW and NDW events are not found in reanalysis products such as the ERA-Interim reanalysis [see Fig. 1 in Karpechko et al. (2017)], but they have been found in large samples obtained from GCMs [e.g., Fig. 3 in Karpechko et al. (2017)]. Hence in Fig. 6 we plot confidence intervals of the DW – NDW difference for the PC stage (−25 to −1) of the NAM index at 700 hPa (Fig. 6a), *F*^(*z*)^ at 700 hPa averaged over 45°–75°N (Fig. 6b), and *Z* at 700 hPa (Fig. 6c) area-averaged over 50°–80°N, 60°–90°E (i.e., the positive differences slightly northwest of the climatological Siberian high). The confidence intervals are estimated using a Monte Carlo repeat sampling procedure (100 000 repetitions) for different prescribed sample sizes. The confidence intervals for the 90% (red), 95% (green), and 99% (blue) levels all converge to the overall composite mean shown in the corresponding figures (see dotted black lines) as the sample size is increased from the minimum of 10 considered here to the maximum of 455. From the definition of a confidence interval around the difference between the means of two samples, if the interval does not contain zero, then the means are significantly different from one another at the chosen level. Hence, we can ascertain from Fig. 6 that the point at which the upper bound crosses the zero difference line to become negative indicates the approximate number of SSWs that are required to obtain the required level of statistical significance (see the respective colored vertical lines).

In terms of the NAM index, it can be seen that at the 90%, 95%, and 99% levels the number of DW SSWs required is ~55, 75, and 115, respectively (in addition to the same number of NDW SSWs). For *F*^(*z*)^, the numbers required are slightly less (~40, 50, and 85), and for *Z* over the Siberian high sector the numbers are slightly less again (~35, 45, and 70). This suggests that the tropospheric precursor that most efficiently discriminates DW from NDW events is the strength of the 700-hPa height anomaly over the Siberian high sector. In all three cases, even at the 90% level, the number of DW and NDW SSWs required separately to find such precursor anomalies is more than double that of the observed number of SSWs in even the JRA-55 reanalysis (which has one of the largest numbers of SSWs among contemporary reanalysis datasets).

### b. Robustness of these precursors

**FIG. 7.**
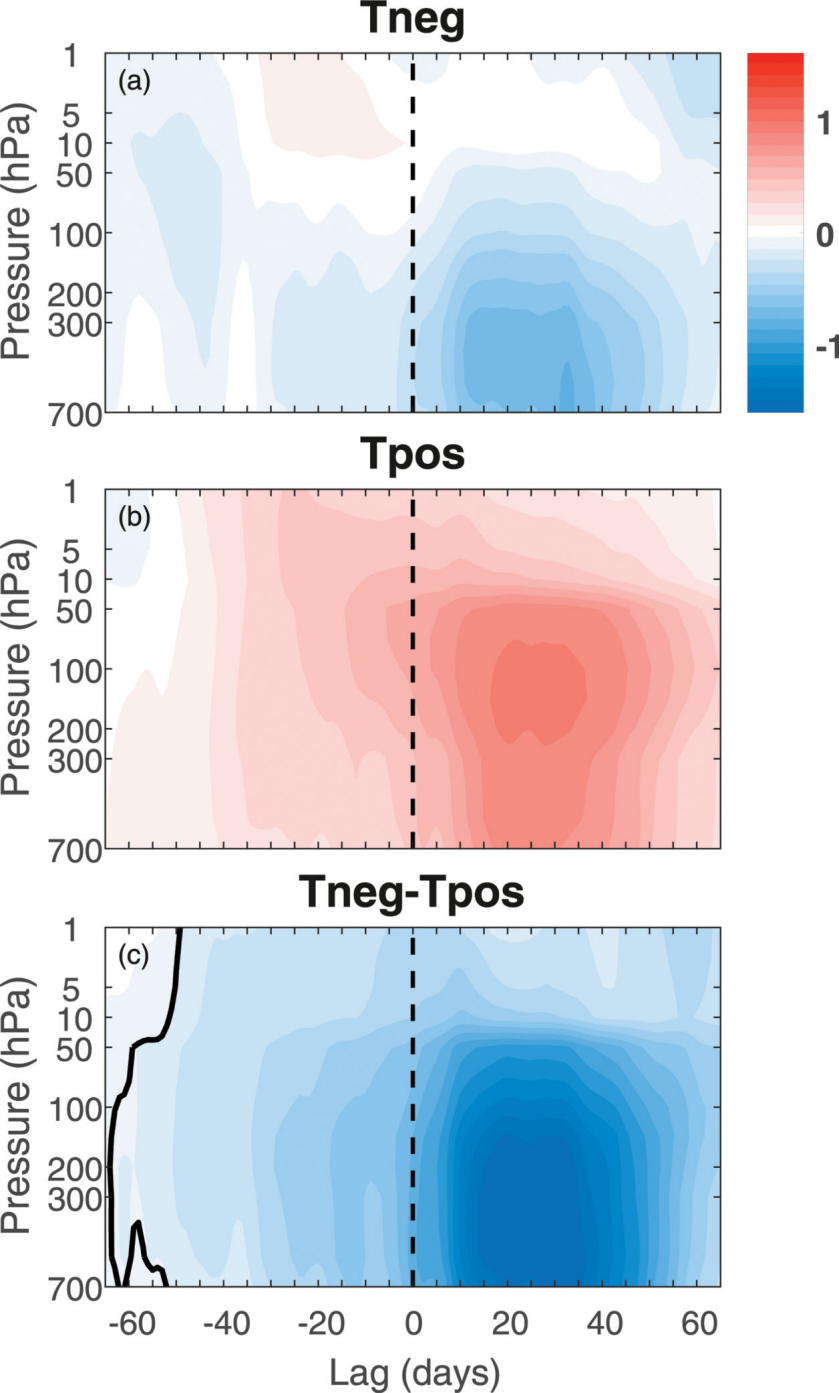
NAM index composited for (a) Tneg and (b) Tpos tropospheric NAM events that have been randomly selected (see text for more details) independent of an SSW influence above. (c) The Tneg – Tpos composite difference; the thick black contour is as in Fig. 1.

The previous section identified tropospheric precursors that appear to distinguish DW and NDW SSWs. We test the robustness of the zonal-mean NAM precursors by comparing the NAM shown in Fig. 1 with that of randomly selected tropospheric events that are independent of an SSW (Fig. 7). The latter allows us to test whether the precursor anomalies to SSWs we have found are simply related to random tropospheric variability. Additionally, we have also tested the robustness to different DW definitions but direct the reader to the online supplemental material for figures and analysis. To calculate this random composite, we removed each SSW event and its surrounding 100 days (hence, 101 days total for each event) from the time series for each experiment, and then randomly selected a new event, which by construction is unrelated to a SSW. We define each event as having a negative (Tneg) or positive (Tpos) tropospheric NAM after the “onset date” by averaging the tropospheric NAM at 500 hPa over lags +10 to +50, yielding 411 Tneg and 551 Tpos events [this is similar to the DW definition of Jucker (2016); see the supplemental information]. By construction, we are sampling only tropospheric internal variability.

While the negative NAM signal in the Tneg composite for positive lags arises by construction (Fig. 7a), the NAM is also negative at negative lags, due to the persistence of the NAM index. The opposite is evident in the Tpos composite (Fig. 7b), although with a larger amplitude. This is due to the fact that the tropospheric NAM index is on average slightly positive when all SSWs are removed. This yields Tneg – Tpos differences that are significantly negative at all lags (Fig. 7c), and which are qualitatively similar to that found in the DW – NDW differences (but with differing magnitudes; compare with Fig. 1d). However, we note that these events are randomly chosen and the onset date has no influence on the tropospheric NAM; indeed, the onset date could be randomly chosen to occur at the start, in the middle, or at the end of the life cycle of the negative tropospheric NAM event, which, when averaged over all 962 events, would conceivably give a composite similar to that shown in Fig. 7. In fact, upon reselecting events hundreds of times, similar composites are found. Nevertheless, this viscerally highlights that the differences at positive lags in the troposphere are entirely there by construction.

**FIG. 8.**
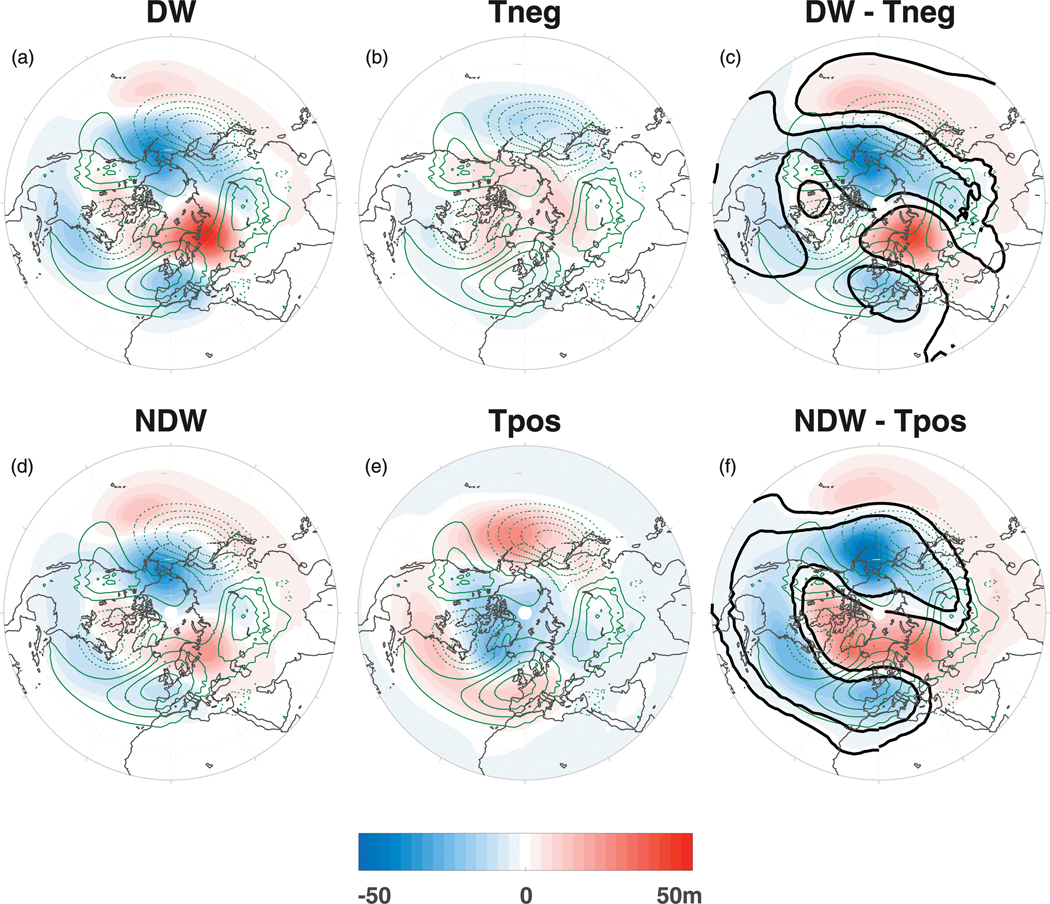
The *Z* anomalies at 700 hPa averaged over the PC stage (lags −25 to −1) for the (a) DW SSWs composite, (b) Tneg events composite, (c) DW – Tneg difference, (d) NDW SSWs composite, (e) Tpos events composite, and (f) NDW – Tpos difference. See Fig. 3 for details on the shading and different contours. Note that (a) and (d) are repeated from Figs. 3a and 3b.

We now examine the latitude–longitude differences between Tneg and Tpos for the random tropospheric events. Figure 8 shows the GPH anomalies at 700 hPa for the DW and NDW SSW events (left column; reproduced from Figs. 3a,b), the Tneg and Tpos events (middle column), and the differences DW – Tneg (right column, top) and NDW – Tpos (right column, bottom). The Tneg events show overall much weaker anomalies than the DW SSW events with negative anomalies at midlatitudes associated with a localized trough over the North Pacific basin and a smaller-valued trough over the North Atlantic basin, and positive anomalies farther poleward. This yields DW – Tneg differences with a high slightly northwest of the climatological Siberian high and a low slightly to the northeast of the climatological Aleutian low, similar to Fig. 3c due to the dominance of the SSW composites. In terms of the Tpos events, there is also a more annular structure, but of opposite sign to the Tneg events, yielding annular and opposite-signed differences to DW–Tneg. The differences between the randomly selected events and the precursor anomalies present in the DW and NDW SSWs at negative lags allows us to conclude that the enhanced wave forcing we have found at the lower levels is a robust feature and not present due to random tropospheric variability.

### c. Relationship between SSW frequency and precursory extreme wave activity

Sections 3a and 3b have demonstrated that in a large composite of SSWs, tropospheric features before the SSW differentiate between SSWs that have a DW impact and those that do not. However, in order to not overstate the importance of tropospheric precursory features evident in such composites, we now examine the spread of individual SSWs and see how many events, both DW and NDW, show evidence of such precursors.

**FIG. 9.**
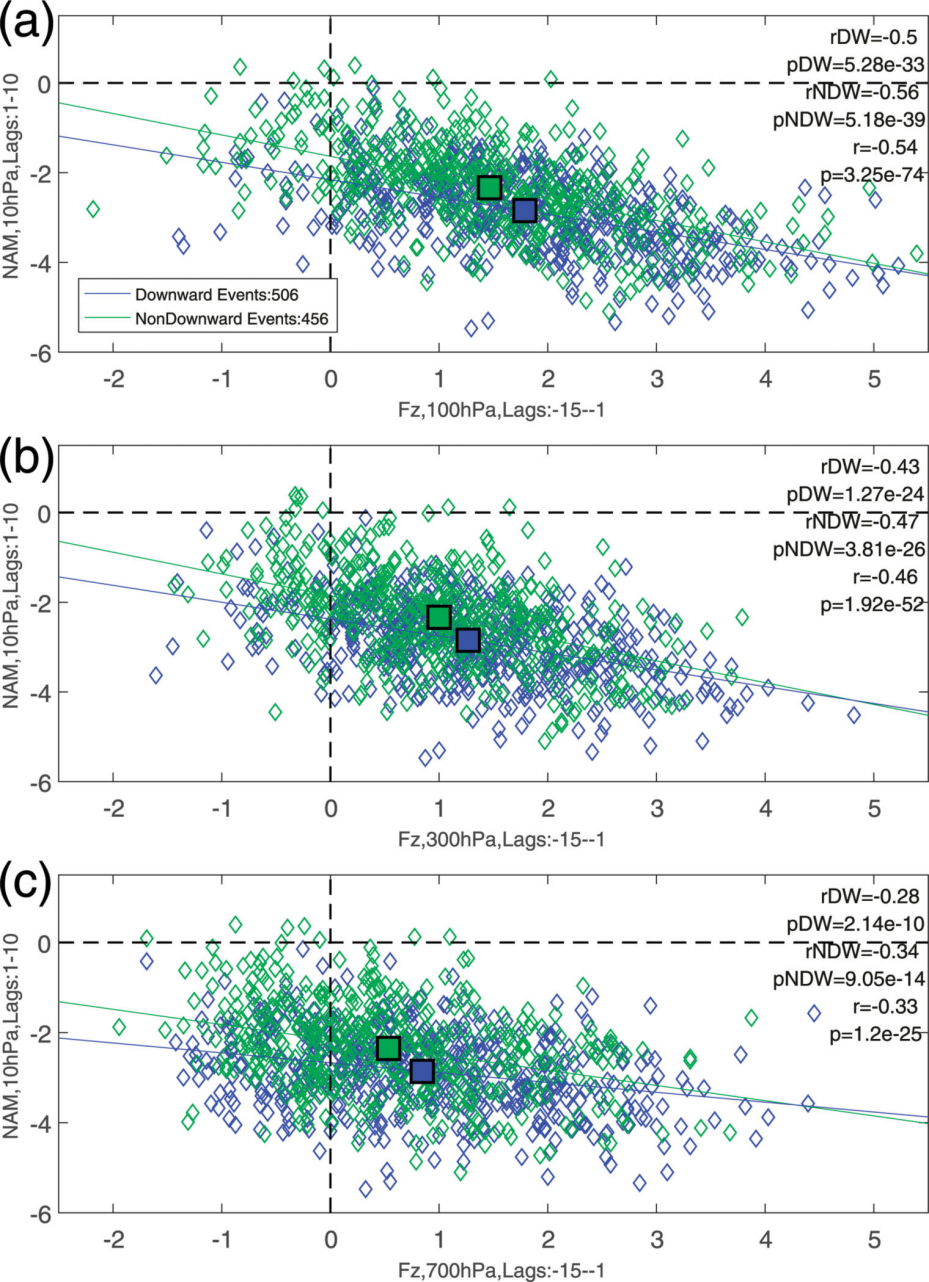
Scatterplots of standardized *F*^(*z*)^ (filtered for wave 1) at (a) 100, (b) 300, and (c) 700 hPa, averaged over lags −15 to −1, against the NAM index at 10 hPa averaged over lags +1 to +10. Blue (green) diamonds, lines, and squares represent, respectively, individual DW (NDW) SSW events, the corresponding lines of best fit, and the overall composite averages. The rDW (pDW), rNDW (pNDW), and *r* (*p*) represent the correlation coefficients and *p* values for the DW events, NDW events, and total, respectively. The values in the top left show the numbers of DW and NDW SSWs that are preceded by such extreme wave activity averaged over lags −15 to −1.

Figures 9a–c shows scatter graphs of *F*^(*z*)^ (filtered for planetary wave 1, averaged over 45°–75°N and standardized as in Fig. 2) at three different levels averaged over lags −15 to −1, against the NAM index at 10 hPa averaged over lags +1 to +10. We note that the patterns are not sensitive to slight changes in the earlier lag for *F*^(*z*)^. The term *F*^(*z*)^ is filtered for wave 1 as this wavenumber appears to play the largest role in the composites shown in Fig. 2. We note that the window for *F*^(*z*)^ used here is shorter than that used in Polvani and Waugh (2004), who found that a time-integrated upward flux over 40 days at 150 hPa gave the best correlation. At all three levels (100, 300, and 700 hPa), the correlation coefficients are negative, indicating that enhanced wave activity gives rise to a weaker polar vortex. However, the overall correlation coefficients are maximized at 100 hPa (−0.54), become weaker at 300 hPa (−0.46), and reduce substantially at 700 hPa (−0.33). At all three levels, the correlation coefficients are statistically significant (*p* < <0:01), which, given the relatively small correlation coefficient at 700 hPa, is likely due to the large sample size. Upon splitting into DW and NDW events, and calculating the lines of best fit for each, it can be seen that the respective correlation coefficients are also both very similar at 100 hPa (−0.50 and −0.56), 300 hPa (−0.43 and −0.47), and 700 hPa (−0.28 and −0.34). The scatter about the lines of best fit, particularly at the lower two levels, is indicative of the high degree of variability in the winter troposphere and stratosphere. The composite mean for both event types (large squares) indicates that for DW events there is a slightly larger upward wave-activity flux at all levels preceding the SSW, which results in a more negative 10-hPa NAM.

The decline in the correlation between the stratospheric NAM and the vertical component of the EP flux as one analyses the EP flux closer to the surface is consistent with the recent papers by Birner and Albers (2017) and also de la Cámara et al. (2017). Specifically, Birner and Albers (2017) found that 25% of SSWs in the relatively short reanalysis record were preceded by extreme lower-tropospheric wave events (LTWEs; 700 hPa). We here further update this statistic using our large ensemble of SSWs. We define a SSW to be preceded by extreme wave activity at a given level if the deseasonalized 11-day running-mean averaged *F*^(*z*)^ exceeds the two-standard-deviation threshold at least once in the preceding 10 days [this 10-day window was found to be appropriate by Sjoberg and Birner (2012) and Birner and Albers (2017)]. This is performed separately for waves 1 and 2, and in order to avoid double counting, if a given SSW event is preceded by both extreme wave-1 and wave-2 fluxes, the wavenumber with the largest *F*^(*z*)^ value is used to define the dominant wavenumber preceding the SSW.

**FIG. 10.**
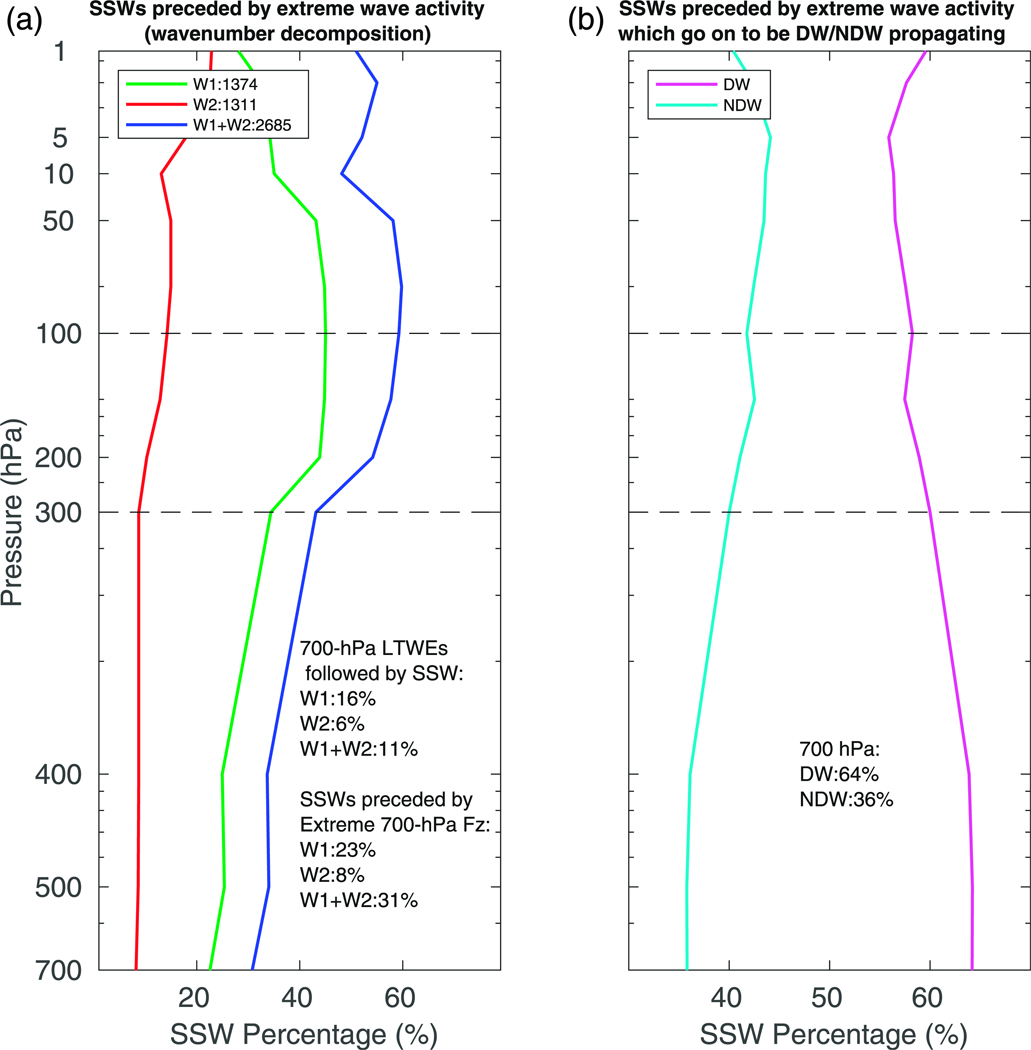
Line plots of (a) the percentage of SSWs that are preceded by extreme (>two standard deviations) *F*^(*z*)^ at each level for wave 1 (green), wave 2 (red), and waves 1 and 2 combined (blue), and (b) the percentages of SSWs that are preceded by extreme wave activity at each level that go on to be DW (magenta) or NDW (cyan) propagating. Inset in (a) are the numbers and percentages of SSWs (rounded to the nearest percent) preceded by lower-tropospheric wave events (LTWEs; 700 hPa) to be compared with Birner and Albers (2017), as well as the numbers of extreme wave-activity events that are followed by an SSW event.

Hence we plot in Fig. 10a the percentage of SSWs that are preceded by extreme upward wave activity as a function of height for wave 1 (green), wave 2 (red), and wave 1 and wave 2 together (blue). The overall profile for wave 1 shows that 45% of SSWs are preceded by at least one day of extreme wave-1 activity at 100 hPa. This figure decreases fairly rapidly with decreasing height with 23% of SSWs being preceded by extreme wave-1 activity at 700 hPa. For wave 2, on the other hand, the percentage of SSWs that are preceded by extreme wave activity at 100 (700) hPa is much smaller than for wave 1, with values of 14% (8%). Perhaps most tellingly, if we combine the two then 31% of SSWs are preceded by extreme wave activity at 700 hPa, which is similar to the 25% observed by Birner and Albers (2017) using ERA-Interim. At 100 hPa, this combined percentage rises to ~60%.

While this result indicates that roughly one-third of SSWs are preceded by extreme wave activity in the lower troposphere, additional insight as to the usefulness of tropospheric wave activity for predicting a SSW can be obtained by examining the number of lower-tropospheric wave events that are followed by SSWs. We define such a LTWE if the 11-day running-mean averaged *F*^(*z*)^ at 700 hPa exceeds the two-standard-deviation threshold during wintertime (October–April). The difference in the number of days between two consecutive LTWEs must be greater than or equal to 10 days. If there is any overlap between any wave-1 and wave-2 events within 10 days, then as before the larger-valued wavenumber is assumed to be dominant. This yields 1374 and 1311 extreme wave-1 and wave-2 LTWEs, respectively.^1^ The percentage of LTWEs that are followed by a SSW is then calculated from the SSWs shown above and the number of LTWEs. The corresponding percentages are inset into the panels in Fig. 10a; 16% (6%) of 700-hPa wave-1 (wave 2) LTWEs are followed by a SSW, together indicating that 11% of LTWEs appear to be followed by a SSW event.

In Fig. 10b, the percentage of SSWs that are preceded by extreme wave activity at each level and that subsequently go on to be either DW or NDW propagating is shown. By construction, the DW and NDW profiles, when summed at each level, equal 100%. The DW profile maximizes in the lower troposphere (below ~400 hPa), suggesting that the presence of extreme wave activity in the lower troposphere appears to be a better indicator of whether the SSW will go on to be DW propagating than such extreme wave activity at higher levels. Indeed, the percentage of SSWs that are preceded by extreme wave activity at 700 hPa and that are subsequently DW propagating is 64% (and conversely 36% for NDW propagation). Hence, in a probabilistic sense, there is a 28% difference between DW- and NDW-propagating SSWs and the tropospheric wave activity that occurs prior to it (consistent with section 3a). However, given that a high percentage of SSWs that are preceded by extreme lower-tropospheric wave activity are NDW propagating, one would not be able to make a deterministic prediction at the onset of whether a given SSW will be DW or NDW propagating.

We note that the same analysis was also performed using the standardized anomalies over the Siberian high sector (50°–80°N, 60°–90°E) at 700 hPa. The percentages were around half of those shown in Fig. 10, with 16% of the total number of SSWs being preceded by such extreme anomalies (greater than two standard deviations). The percentage of SSWs preceded by such anomalies that then go on to be DW (NDW) propagating is 62% (38%). Hence despite Fig. 6 indicating that examining the GPH anomalies over the Siberian high sector may be a more robust way to examine the DW influence of SSWs, these percentages indicate that instead *F*^(*z*)^ may be a better indicator.

### d. Precursors to splits and displacements

So far we have only focused on the precursors to SSWs identified using the CP07 approach. Here we examine the precursors associated with splits and displacements identified using the method of S13. Additionally, in light of recent studies that have found differing results with regards to which type of event has the most noticeable surface impact after the onset date (Mitchell et al. 2013; S13; Maycock and Hitchcock 2015), we again use the DW definition of Karpechko et al. (2017) to examine the DW influence of both splits and displacements.

**FIG. 11.**
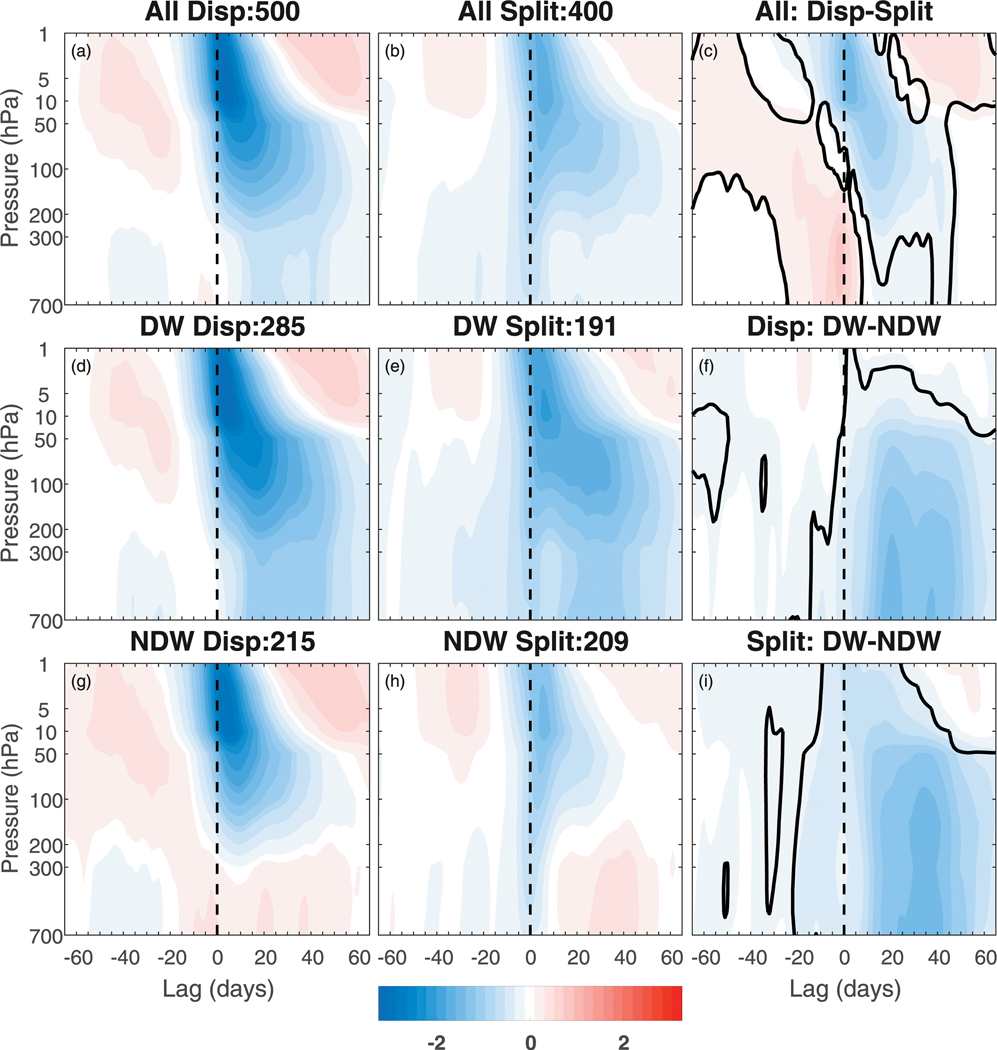
Composite evolution of the NAM index divided into (left column) displacements and (middle column) splits, and subdivided further into (a),(b) total, (d),(e) DW-propagating and (g),(h) NDW-propagating SSWs. The right column shows the (c) displacements – splits, (f) DW – NDW displacements, and (i) DW – NDW splits. See Fig. 1 for further details on shading and different contours.

Figure 11 shows the height–time evolution of the NAM index divided into displacements (left column) and splits (middle column) and subdivided further into the total (top row), DW-propagating (middle row) and NDW-propagating (bottom row). Also shown are the differences (right column) for displacements and splits (top), DW and NDW displacements (middle), and DW and NDW splits (bottom). In the total composites, clear significant differences between displacements and splits can be seen in both the stratosphere and in the troposphere. In the stratosphere, the displacements are stronger than the splits, up until lag +50. In particular, in the middle-to-upper stratosphere the displacements are nearly twice as strong. In the troposphere, while the displacement events have a stronger long-term influence up until lag 145, the splits have a more barotropic nature at the onset with an instantaneous response near the surface, which dissipates after ~lag +5. The barotropic nature at the onset is in agreement with the more likely role of the barotropic mode for split SSWs (Esler and Scott 2005). Prior to the onset date, the splits show clear tropospheric negative anomalies extending back to lag −45, which are stronger than for the displacements.

Upon subdividing into DW (middle row) and NDW (bottom row) events, the splits and displacements broadly show similar results to those found using the wind reversal criterion (Fig. 1), with slightly stronger negative NAM anomalies in the middle to upper stratosphere as well as longer-persisting anomalies in the lower stratosphere for DW events. This therefore yields similar DW – NDW composite differences at positive lags to Fig. 1. However, at negative lags, the splits have much stronger negative tropospheric and lower-stratospheric precursors than the displacements, extending back to lag −55 and becoming stronger around lag −25 for the DW events, but weaker anomalies extending back to lag −30 for the NDW splits. The DW displacements, on the other hand, show very similar anomalies to the total (Fig. 11a), and the NDW displacements show evidence of positive tropospheric anomalies up to two weeks before the onset (and weakly negative anomalies before that). Overall, this gives similar-valued DW – NDW differences at negative lags, except that the splits have negative differences that extend farther back to lag −30 and also extend into the stratosphere.

**FIG. 12.**
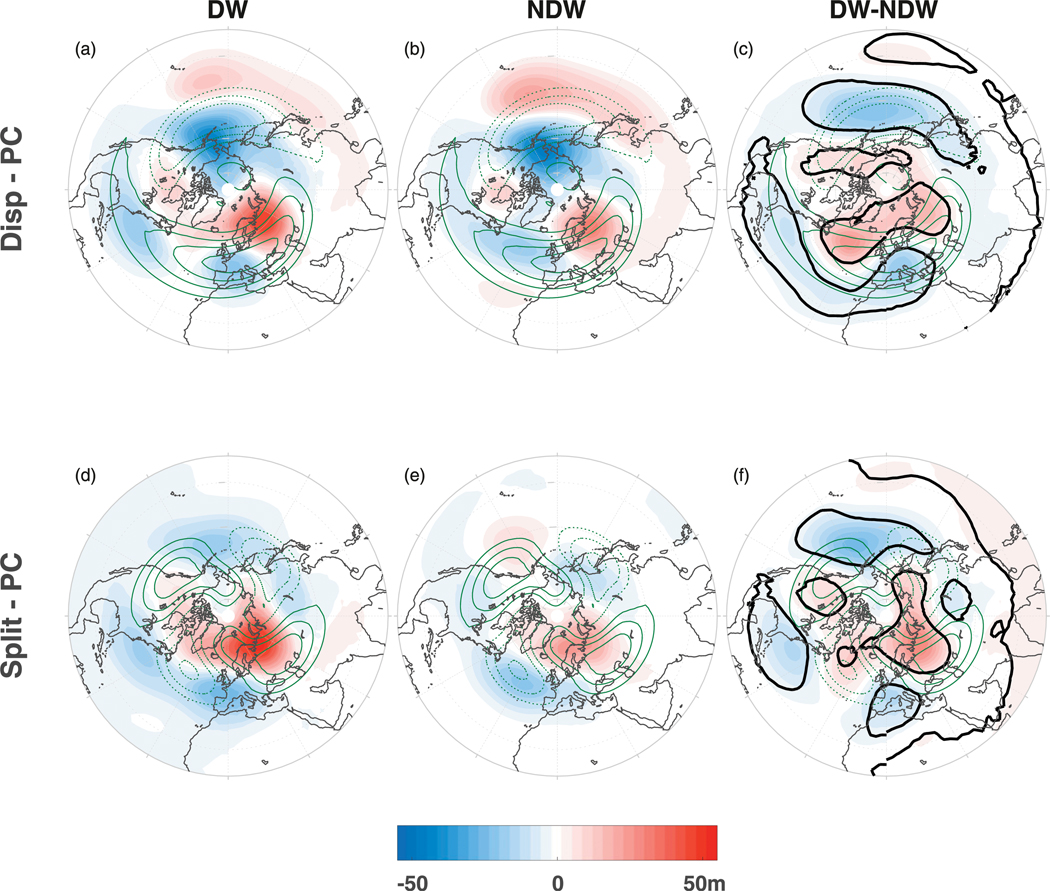
As in top row of Fig. 3, but for *Z* at 700 hPa during the PC stage for (a)–(c) displacement SSWs and (d)–(f) split SSWs. Note that the green contours show the climatological *Z* filtered only for (top) wave 1 and (bottom) wave 2 and with a contour interval of 10 m.

As before, we now examine the regional differences in order to understand these tropospheric precursors. Figure 12 shows the same as the PC anomalies in Fig. 3 except for *Z* at 700 hPa for the (top) displacement and (bottom) split events. Note that we do not show the ONS and REC stages in this plot as they are similar to those in Fig. 3. For the displacement events, there are negative anomalies over the northwestern Pacific and positive anomalies over northern Europe and Siberia. These two anomalous centers project onto the climatological wave-1 centers of action (green contours); in particular, the positive anomaly over northern Europe and Siberia is more positive for the DW events, indicating, similarly to Fig. 3, an increase in upward wave 1. Also over the subtropical North Pacific there is a band of positive anomalies projecting onto the eastern flank of the climatological wave-1 Aleutian low. These anomalies are more positive under NDW events and hence yield negative differences over the Aleutian low sector. This subtropical band of positive anomalies in conjunction with the negative anomalies farther poleward yields a dipole over the Pacific basin leading to possible meridional shifts in the east Pacific jet (e.g., Nishii et al. 2010; Dai and Tan 2016; Bao et al. 2017).

For the split events (bottom), the anomalies at this level show more of a wave-2 structure, with an intensification of the highs and lows of the climatological wave 2 (green contours). In particular, there are negative anomalies over the North Pacific, the North Atlantic, and western Europe, along with positive anomalies over Siberia and eastern Europe. In general, these anomalies are stronger for the DW events, as indicated by the difference composite. The differences also show evidence of an intensification of the climatological wave 1.

**FIG. 13.**
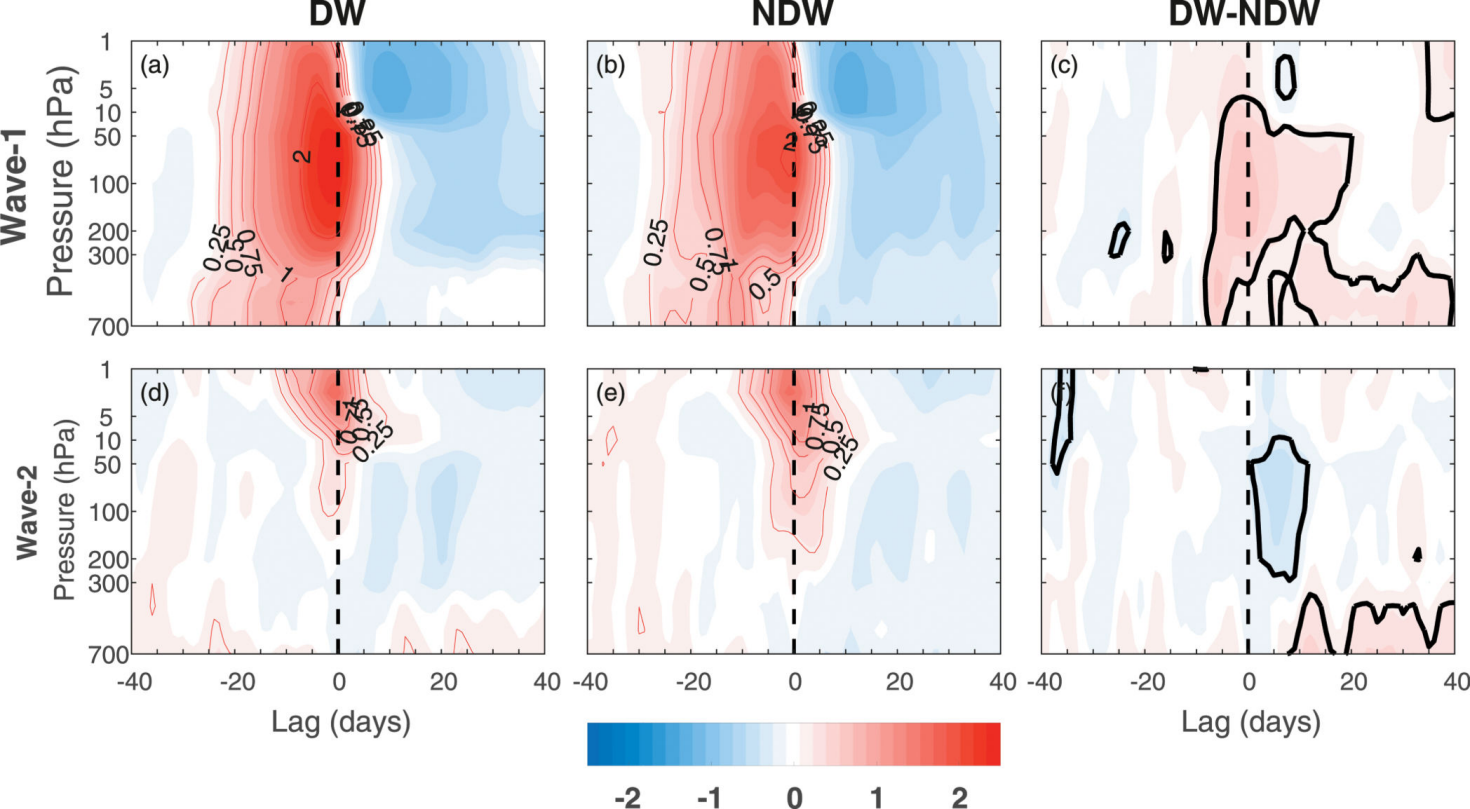
Height–time plot of *F*_*z*_ averaged over 45°–75°N for the displacement SSWs composited over (left) DW events and (middle) NDW events, and (right) the DW – NDW difference, for *F*_*z*_ for (a)–(c) wave 1 and (d)–(f) wave 2. The thick black contour in the difference plots represents statistical significance at the 95% level.

**FIG. 14.**
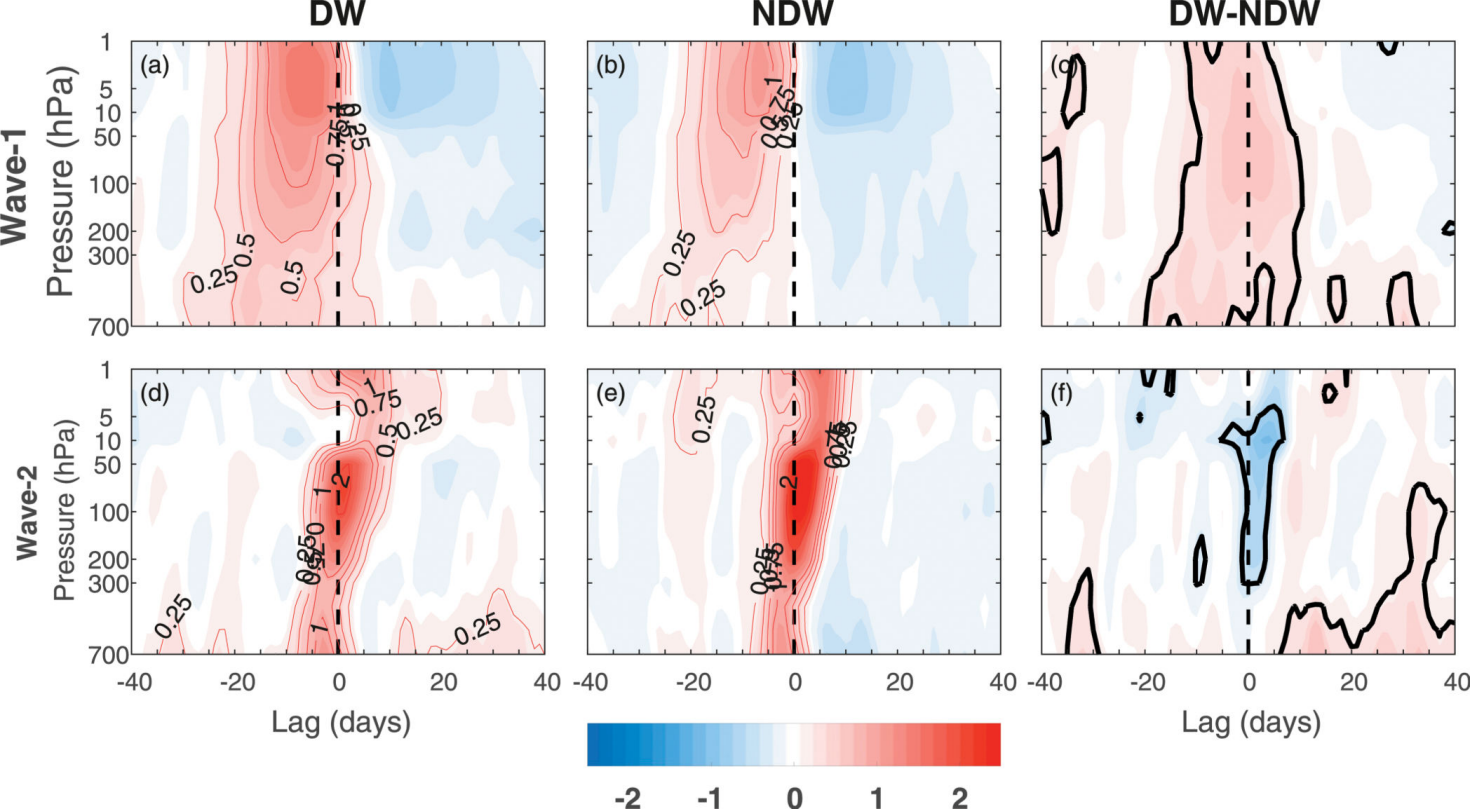
As in Fig. 13, but for split SSW events

We now plot the height–time evolution of *F*^(*z*)^ for displacement events (Fig. 13) and split events (Fig. 14) in order to determine the vertical extent of the wave-1 (top row) and wave-2 (bottom row) anomalies from the troposphere into the stratosphere. As in Fig. 2, the anomalies are standardized by their standard deviation at each pressure level. For the displacements, the wave-1 anomalies are generally similar to those in the wave 1 and 2 composite shown in Fig. 2. For DW events, there is enhanced upward wave 1 compared to NDW events, which propagates up from 700 hPa into the stratosphere peaking close to the onset date. After the onset, the wave activity is generally suppressed as shown by negative anomalies in both the DW and NDW events, although positive (upward) anomalies do persist in the upper troposphere to lower stratosphere for ~5–10 days after the onset. The negative anomalies for the NDW events are of significantly larger magnitude. Note that the other wavenumbers contribute negligibly to the *F*^(*z*)^ flux and hence we do not include them here, for brevity.

For split events (Fig. 14), we can see that they are generally preceded by upward wave-1 and wave-2 anomalies that propagate up from 700 hPa and peak in the stratosphere. As in the displacements, the standardized anomalies are larger in the stratosphere than in the troposphere. This is the case for both DW and NDW events, although there is actually slightly less upward wave 2 at the onset for the DW events [Fig. 14f; opposite to Nakagawa and Yamazaki (2006)]. However, those that propagate DW to the troposphere are on average preceded by enhanced anomalous upward wave 1 into the stratosphere (Fig. 14c). In the wave-1 difference (Fig. 14c) it can be seen that this enhanced upward wave 1 for DW events starts around lag −20 and persists through the onset date until around lag +10. Even though split events are generally associated with wave-2 anomalies in the upward flux (as shown in Figs. 14d,e), this result indicates that wave 1 may also play a role in the DW influence. Similar to the displacements, there are enhanced upward tropospheric wave-2 anomalies for the DW events after the onset date.

## 4. Summary and discussion

Using a series of 40 integrations of the GEOSCCM model, we have 1) identified and analyzed the frequency of tropospheric precursory features to SSWs (generally, and for splits and displacements) that appear to manifest as zonally varying wave patterns that project onto the climatological stationary planetary centers, extending the recent observational study of Birner and Albers (2017); and 2) examined the differences in such precursors between so-called downward (DW) and non-downward (NDW) propagating SSWs. To do this we identified a large compendium of SSWs across all 40 runs using the definition of Charlton and Polvani (2007). This yielded a ratio of approximately 0.61 SSWs per year (~950 in ~1600 years), which were then classified as DW and NDW-propagating using a variety of recently developed DW definitions (Jucker 2016; Runde et al. 2016; Karpechko et al. 2017).

For the SSWs in general, there is an enhanced upward flux of wave activity into the stratosphere from the troposphere preceding the SSW onset. In a composite sense, the enhanced wave activity appears to originate in the lower troposphere (Figs. 2–5, 13, and 14), although relative to its local standard deviation, the anomalies in the stratosphere are at least twice as large as those in the troposphere, in agreement with similar composites in Jucker (2016) and Birner and Albers (2017). This occurs as a projection of the anomalies onto the climatological centers of action, associated with a deepening of the Aleutian low and a strengthening of the Siberian high and yielding an enhanced upward wave-1 flux. The enhancement of upward wave-1 activity prior to the onset followed by the subsequent reduction at later times is in agreement with the observational composites of Limpasuvan et al. (2004) using reanalysis data.

Recent studies by Jucker (2016), Birner and Albers (2017), and de la Cámara et al. (2017) found that anomalous upward fluxes of lower-tropospheric wave activity were not a necessary or sufficient precursor to SSW events, given that only one-quarter of SSWs in the period covered by ERA-Interim were preceded by such wave events. Instead, they found that the state of the stratosphere prior to the onset date played a much more important role in determining the occurrence of an SSW. The stratospheric state may be in a preferable configuration to take advantage of the climatologically large tropospheric reservoir of wave activity and encourage an anomalous upward wave flux across the tropopause. Our results in section 3c agree well with the results of Birner and Albers (2017), despite the shortness of the observational record, as 31% of SSWs are here found to be preceded by extreme lower-tropospheric (700 hPa) wave activity (Fig. 10).

The number of SSWs that were preceded by extreme wave activity increases rapidly up to 100 hPa (~60%). Given that at high latitudes the 100-hPa surface is already well within the vortex (de la Cámara et al. 2017), this is perhaps expected. Furthermore, the correlations between the vertical wave flux (which is again maximized at 100 hPa) and the strength of the polar vortex at 10 hPa reduce substantially closer to the surface (Fig. 9). This is indicative of the fact that even in the presence of lower-tropospheric wave activity, the high degree of internal atmospheric variability can easily prevent such wave activity from propagating upward into the stratosphere. Indeed, it still remains to be seen how even in the presence of extreme tropospheric wave fluxes the stratosphere can (or cannot) take advantage of such anomalous wave fluxes. However, our study cannot shed light on the ingredient that allows for this.

In the case of DW-propagating SSWs, we find evidence of both significantly enhanced zonal-mean and regional tropospheric precursors, compared to the NDW SSWs in the composites shown in Figs. 1–5. In terms of the zonal mean, negative NAM anomalies were found to exist throughout the troposphere prior to the onset date for DW events, with negative DW–NDW differences extending as far back as lag −40 (see Fig. 1). NAM precursors were also found previously using large numbers of simulated SSW events (e.g., Jucker 2016; Karpechko et al. 2017). However, as aforementioned, such NAM precursors have been shown to be both model- and configuration-dependent (Gerber et al. 2010). This is consistent with Black and McDaniel (2004), who observed that the determination of the DW propagation of a SSW depended on the pre-existing tropospheric state, with a pre-existing positive NAM-like state being associated with NDW SSWs, and vice versa. Note that using three of the four recently proposed DW definitions (Runde et al. 2016; Jucker 2016; Karpechko et al. 2017) yields similar precursory features (see the online supplemental information for details and a discussion of the fourth definition, which yields different results).

Further, enhanced upward zonal-mean wave-activity fluxes *F*^(*z*)^ were also found (Fig. 2) to precede DW SSWs extending back to around lag −25. These standardized anomalies spanned the depth of the troposphere and intensified in the stratosphere above 200 hPa. By splitting the SSWs according to the magnitude of the *F*^(*z*)^ anomalies prior to the onset date rather than according to the magnitude of the NAM after the onset, it was found that, on average, those events with larger *F*^(*z*)^ led to a more negative tropospheric NAM signal after the onset (Fig. 4).

In a regional sense, there appear to be differences between DW- and NDW-propagating SSWs in the geopotential height in the troposphere and lower stratosphere (Figs. 3–5 and 8), which strengthen the wave anomalies already associated with the onset of the SSW. The regional differences are particularly large over northern Europe and Siberia, with a strengthening of the climatological Siberian high under DW events. We note that such anomalies over the Siberian high sector prior to DW-propagating SSW events were also found in observations by Nakagawa and Yamazaki (2006) using the 45-yr ERA-40 reanalysis dataset.

Previous work has showed a wide disparity in the sign of the tropospheric NAM signal before SSWs [see Fig. 10 of Gerber et al. (2010)]. With the availability of 900+ SSWs, we more clearly see this negative NAM precursor, although at least 55 DW and 55 NDW events are needed before this NAM feature becomes robust [Fig. 6; note, however, that only 35–40 DW and NDW events separately are required to find robust differences in *F*^(*z*)^ and *Z*]. Indeed, in only a handful of the individual members of the 40-member ensemble are such tropospheric NAM precursors present (not shown), suggesting that the diversity evident in Gerber et al. (2010) arises not only from peculiarities of the various models but also from internal variability. Note that this is also in agreement with the work of Gerber et al. (2009) and Hitchcock and Simpson (2014), who suggested that the tropospheric response to a SSW consisted of a forced tropospheric component (by the SSW) and a stochastic component that is independent of the SSW above. Indeed, in their runs, they found that a given SSW event may or may not influence the troposphere depending on tropospheric natural variability, which can act to mask any actual DW stratospheric signal. As our analysis indicates that at least 55 SSWs of each type are required before the NAM-precursor effect becomes salient, it shows that internal tropospheric variability can indeed mask any forced signal from the stratosphere. Nevertheless, our results also indicate that the forced signal from the stratosphere is stronger on average if the precursory wave flux from the troposphere is stronger.

Examining the numbers of SSWs that are preceded by extreme lower-tropospheric wave activity and go on to be DW or NDW propagating gives an idea as to how useful such precursory wave activity may be in predicting the tropospheric impact following a SSW. Indeed, of the 296 SSWs that were preceded by such wave activity, 64% (36%) subsequently went on to be DW (NDW) propagating. This enhances the probabilistic prediction of tropospheric impacts following a SSW as it suggests that if a given SSW was preceded by extreme lower-tropospheric wave activity, then one could say at the onset that there is a greater likelihood that it will propagate DW to the troposphere. However, given that a relatively high percentage of SSWs were also preceded by such wave activity and went on to be NDW propagating, one would not be able to make a deterministic prediction before the onset of whether a given SSW will be DW or NDW propagating. Nevertheless, these percentages augment themselves with similar percentages shown in Karpechko et al. (2017, see their Fig. 5), whose results suggested that the likelihood of a SSW having a DW tropospheric impact depends on the sign and magnitude of the lower-stratospheric NAM index and *F* ^(*z*)^ just after the onset date; in particular, the more negative the 150-hPa NAM is at lags 0–4 following the SSW, the more likely it is to propagate DW at later lags.

We also compared the results to those obtained using composites of randomly selected tropospheric events, which by construction were chosen to be unrelated to the SSW above (see section 3b). In a zonal mean, the composites for the DW and NDW SSWs and for the negative (Tneg) and positive (Tpos) random tropospheric events were remarkably similar at all lags (Fig. 7), albeit with changes in magnitude. The replicability of the tropospheric zonal-mean NAM at both positive and negative lags using random events based solely on the behavior of the troposphere suggests the need for caution when just using the NAM to examine the DW influence of a SSW event, as it can conceal much of the regional information that is important for understanding the precursors.

However, the regional precursors, which were found to be associated with upward planetary wave-1 forcing for the SSW events, were very different for the random composites, instead having a weak annular structure (Fig. 8). Because of the differences in the regional tropospheric precursory features between SSW events and randomly selected events, we conclude that the precursors here found are robust and that there is a difference prior to DW and NDW SSWs other than just random tropospheric variability.

The converse to examining the proportion of SSWs (either DW or NDW propagating) that are preceded by extreme lower-tropospheric wave activity is to consider the proportion of such events that are followed by a SSW within 10 days. In total, 11% of the identified lower-tropospheric wave events (16% of wave 1 and 6% of wave 2) were followed by an SSW. Despite this figure being twice as large as the observed 6% of tropospheric blocks that are followed by a SSW event in 44 years of reanalysis data (Martius et al. 2009), we stress that it is impractical to forecast SSWs based solely on identifying extreme tropospheric wave events (e.g., Birner and Albers 2017).

We finally examined the evolution of the troposphere and stratosphere associated with split and displacement SSW events. We found that 1) displacements tend to have a longer-term tropospheric influence, and 2) splits have a more barotropic influence at the onset date (Fig. 11). The former is in agreement with Maycock and Hitchcock (2015) using a large sample of SSWs from a long model integration and the method of Seviour et al. (2013) to classify events. However, their results were not robust as using a different classification method yielded different results. Regarding split SSWs, the barotropic influence is in agreement with the barotropic mode leading to a split SSW (Esler and Scott 2005; Matthewman et al. 2009; Seviour et al. 2016). However, these results overall disagree with studies by Mitchell et al. (2013), Seviour et al. (2013), O’Callaghan et al. (2014), and Lehtonen and Karpechko (2016), who found that splits have a larger tropospheric influence than displacements in reanalysis data lasting up until lag 160. The disagreement may be related to the differences in sample sizes, which is an order of magnitude larger in our study. Indeed, we created composites for each individual experiment (not shown), and in a handful of the 40 ensemble member, composites are qualitatively similar to Mitchell et al. (2013). However, we note that our results are more in agreement with Seviour et al. (2016), who used 13 stratosphere-resolving models from the ensemble from phase 5 of the Coupled Model Intercomparison Project (CMIP5) and found that despite splits exhibiting a slightly stronger signal over the North Atlantic for upto one month after the SSW, the largest and most significant differences were associated with displacements over Siberia. We note that our results therefore are also slightly in disagreement with Karpechko et al. (2017), who in their large ensemble of SSWs obtained from a chemistry–climate model instead found indistinguishable differences between the two types of events.

We also found that in general, the splits and displacements were associated with enhanced upward wave-2 and wave-1 forcing, respectively (Figs. 13 and 14; e.g., Andrews et al. 1987; Nakagawa and Yamazaki 2006; Liu et al. 2014; Lehtonen and Karpechko 2016) extending into the middle-to-lower troposphere, although we note that there was enhanced wave 1 present for both types. Further, those splits and displacements that propagate DW to the troposphere were associated with even further enhanced wave-1 fluxes at negative lags as compared to NDW-propagating events. The enhanced wave-2 forcing for the splits was more barotropic, occurring closer to the onset date, than for the enhanced wave-1 forcing. The near-barotropic wave-2 nature closer to the onset in association with the larger percentage of SSWs being preceded by extreme lower-tropospheric wave-1 rather than wave-2 fluxes (Fig. 10) suggests that split SSWs may be more nonlinear and thus potentially more difficult to predict.

The results in this paper indicate that the strength of the wave forcing both prior to and during the SSW onset and the subsequent strength of the SSW may play a role in the DW influence of the SSW. However, as mentioned previously, the results only show evidence of an enhancement in probabilistic forecasts of the DW influence; deterministically one could not say if a given SSW event will have such an influence. Hence, given the statistical nature of our analysis, we cannot establish whether the precursor patterns associated with DW-propagating SSWs identified here play a causal role in the tropospheric impact. As this paper only focuses on the output from a single model, future work using observations and/or integrations using different models is required to determine whether the enhanced wave-1 activity and zonal structure of the precursors (e.g., the enhanced Siberian high) play a role in the mechanism, and if so, how.

## Supplementary Material

SUPP

## Figures and Tables

**TABLE 1.** Table showing the number of SSWs according to the two main SSW definitions used in this study: the reversal of *ū* at 60°N and 10 hPa (CP07), and the 2D vortex moments to identify split and displacement (disp) events (S13). Also included are the total number of DW and NDW SSW events calculated using the definitions of Karpechko et al. (2017), Runde et al. (2016), and the absolute-criterion and relative-criterion definitions of Jucker (2016). See text for further details.

	Total			DW			NDW		
Method	Split		Disp	Split		Disp	Split		Disp

			Karpechko et al. (2017)						
CP07 wind reversal		962			506			456	
S13 2D moments	400		500	191		280	209		220
			Runde et al. (2016)						
CP07 wind reversal		962			418			544	
S13 2D moments	400		500	148		239	252		261
			Jucker (2016): Absolute criterion						
CP07 wind reversal		962			370			592	
S13 2D moments	400		500	135		190	265		310
			Jucker (2016): Relative criterion						
CP07 Wind reversal		962			536			426	
S13 2D moments	400		500	187		288	213		212

## Footnotes

1 We note that this definition is slightly different from that used in Birner and Albers (2017), who define a start and end date for an LTWE as the first exceedance of two standard deviations and the subsequent first drop below two standard deviations, respectively. Then, no other LTWE can be defined in the 20 days following the end date (T. Birner, 2018, personal communication). An SSW is determined to follow the LTWE if it occurs within 10 days of the end date. Nevertheless, our results are insensitive to this definition, as in our analysis this definition yields 2626 (1338 wave 1 and 1288 wave 2) independent LTWEs, with 27% of SSWs being preceded by a LTWE in this way (cf. 31%).
