# Supplementary material for: The Downward Influence of Sudden Stratospheric Warmings: Association with Tropospheric Precursors
Source: J Clim. Author manuscript; Available in PMC 2020 Aug 20. (PMC7440399; doi:10.1175/JCLI-D-18-0053.1)
Supplement: SUPP [file NIHMS1531031-supplement-SUPP.pdf]

Supplement to: The Downward Influence of Sudden Stratospheric Warmings: Association with Tropospheric Precursors

Ian White*

The Hebrew University of Jerusalem, Institute of Earth Sciences, Edmond J. Safra Campus, Givat Ram, Jerusalem, Israel

Chaim I. Garfinkel

The Hebrew University of Jerusalem, Institute of Earth Sciences, Edmond J. Safra Campus, Givat Ram, Jerusalem, Israel

Edwin P. Gerber

Courant Institute of Mathematical Sciences, New York University, New York, USA

Martin Jucker

School of Earth Sciences, The University of Melbourne, Parkville, Australia

Valentina Aquila

American University, Dept of Environmental Science, Washington, DC, USA

Luke D. Oman

NASA Goddard Space Flight Center, Greenbelt, Maryland, USA

Corresponding author address: Ian White, The Hebrew University of Jerusalem, Institute of Earth Sciences, Edmond J. Safra Campus, Givat Ram, Jerusalem, Israel.

E-mail: ian.white@mail.huji.ac.il

ABSTRACT

1. Other Downward-Propagating Definitions

We here provide a more detailed description of the three extra definitions of downward (DW) propagating SSWs used in this study of which the numbers are summarised in table 1 in the main text; one by Runde et al. (2016) and two by Jucker (2016):

Runde et al. (2016)

They proposed a more restrictive definition than that by Karpechko et al. (2017). In particular, the NAM index has to be more negative than -1.5 standard deviations at every level below 10 hPa down to 850 hPa for at least one day in the succeeding 70 days (although we chose this window) after the onset date. Additionally, the date of the first exceedance of the threshold at each level must be after (or occur simultaneously to) the first exceedance at the level above. If this is not satisfied then the end date of the exceedance at a given level must occur after (or again simultaneously to) the end date at the level above. Further, the start lag of the threshold exceedance at a given level must be within 30 days of the end date of threshold exceedance at the level above, to try and ensure that the anomalies at each level are connected (personal communiation). Overall this ensures that there is a seemingly DW propagation from the middle stratosphere to the lower troposphere.

Jucker (2016)

The two proposed definitions by Jucker (2016) are referred to here and in the main text as the absolute-criterion and relative-criterion definitions. The absolute-criterion definition simply demands that the NAM index averaged over lags +10 to +40 be smaller than -0.6. We note, as they do, that our results are insensitive to changes in this window, as well as changes in the threshold value. On the other hand, the relative-criterion definition demands that the relative change of the

NAM index at 500 hPa between positive lags (averaged over lags +1 to +80) and negative (averaged over lags -80 to -1) must be smaller than -0.1. We note again, that the results are not sensitive to the thresholds in this definition, aside from the fact that the averaging periods used influence the width of the positive and negative anomalies either side of the onset date in the composite plots.

One thing to be mindful of when identifying a given SSW as DW-propagating is to determine if the tropospheric NAM anomalies are actually attributable to those in the stratosphere. More specifically, the negative tropospheric NAM at positive lags could be due to either the stratospheric anomaly propagating DW, or, due to the persistence of a negative tropospheric NAM prior to the onset. Indeed, it could also be a combination of the two, or even the negative tropospheric NAM at positive lags spontaneously developing, unrelated to the stratosphere. Of course, to distinguish between all of these is very difficult, but from our sensitivity tests, the definitions by Karpechko et al. (2017) and Runde et al. (2016) go some way towards ensuring this, with particular emphasis on the latter which demands an apparent systematic DW propagation from the middle stratosphere to near the surface. Note that the Karpechko and Runde definitions yield quantitatively similar results, and because the former gives a larger compendium of DW SSWs (see table 1 in the main text), we choose to mostly utilise the definition by Karpechko et al. (2017) there, unless explicitly stated otherwise.

2. Robustness of Precursors to DW Definition

We now test the robustness of the zonal-mean NAM precursors using each of the DW definitions introduced above. Figure 1 shows the NAM index at 500 hPa for the DW definition of Karpechko et al. (2017) (red line; see also figure 1 in main text), Runde et al. (2016) (blue

line), and the absolute- and relative-criterion definitions of Jucker (2016) (green and black lines, respectively). We first note that at positive lags, all definitions show negative NAM for DW events by construction, although with differing magnitudes depending on the thresholds used in the individual definitions. At negative lags, the Karpechko, Runde and absolute-criterion definitions give quantitatively similar results to one another, with the DW composite showing negative NAM values prior to lag zero, and the NDW composite showing positive values from approximately lag -20 to 0 and negative values beforehand. This gives differences that are therefore negative and statistically significant extending back to approximately lag -25.

The relative-criterion definition gives drastically different results however for the DW and NDW composites prior to lag zero; positive anomalies for DW events and negative anomalies for NDW events, yielding positive differences prior to lag zero. The differences are antisymmetric (although the negative NAM at positive lags is of larger magnitude) around the central date and this is found to depend on the averaging window used to determine the DW propagation; in this example we used lags -40 to -10 and lags +10 to +40 as the averaging periods. This also agrees with Jucker (2016) who showed a similar composite centred on lag zero.

The differences in the NAM evolution among the four definitions can be related to the periods of time used in each definition. For instance, the Karpechko, Runde, and absolute-criterion definitions only use values of the NAM at positive lags, whereas the relative-criterion uses NAM values at both negative and positive lags. In regards to the former three, they can be used to identify possible precursor features at negative lags (and in fact, the Karpechko definition can be used up until lag +7) as required for this study, as they do not force the composites at such lags. In the case of the relative-criterion definition however, any precusors may be influenced by the

definition. For this reason, we believe that the presence of the precursors are robust but we note that they are sensitive to the type of definition used.

References

Jucker, M., 2016: Are sudden stratospheric warmings generic? insights from an idealized gcm. *J. Atmos. Sci.*, **73**, 5061–5080.

Karpechko, A. Y., P. Hitchcock, D. H. W. Peters, and A. Schneidereit, 2017: Predictability of downward propagation of major sudden stratospheric warmings. *Q. J. R. Meteorol. Soc.*, **143**, 1459–1470.

Runde, T., M. Dameris, H. Garny, and D. E. Kinnison, 2016: Classification of stratospheric extreme events according to their downward propagation to the troposphere. *Geophys. Res. Lett.*, **43**, 6665–6672.

LIST OF FIGURES

Fig. 1. NAM index at 500 hPa composited over (a) DW events, (b) NDW events, and (c) DW-NDW differences, for the four DW definitions introduced previously: red, dark blue, green and black lines represent the NAM index for the Karpechko et al. (2017) definition, Runde et al. (2016) definition, and absolute- and relative-criterion definitions of Jucker (2016), respectively. There is also an additional cyan line representing the NAM index found using a random selection of tropospheric NAM events (see main text). The thick lines represent statistical significance at the 95% level. 9

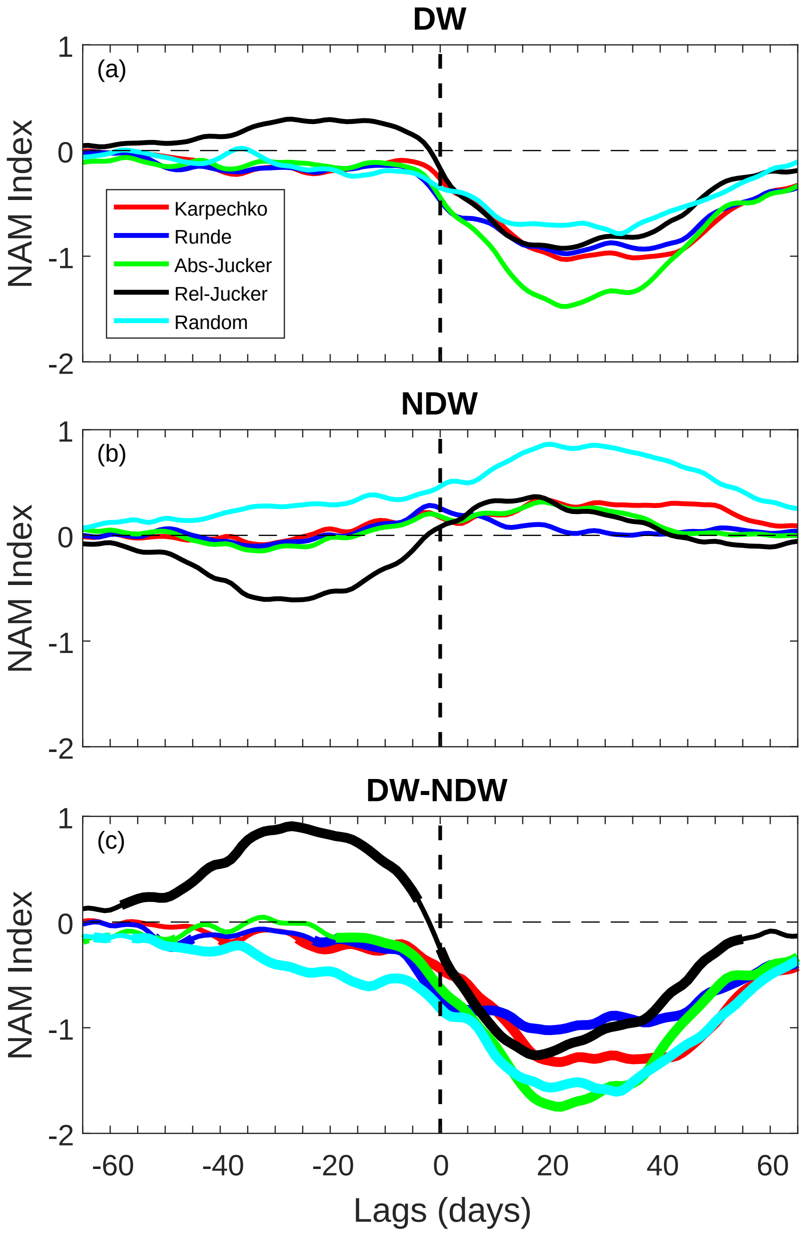

FIG. 1. NAM index at 500 hPa composited over (a) DW events, (b) NDW events, and (c) DW-NDW differences, for the four DW definitions introduced previously: red, dark blue, green and black lines represent the NAM index for the Karpechko et al. (2017) definition, Runde et al. (2016) definition, and absolute- and relative-criterion definitions of Jucker (2016), respectively. There is also an additional cyan line representing the NAM index found using a random selection of tropospheric NAM events (see main text). The thick lines represent statistical significance at the 95% level.
